# NG2 glia regulate brain innate immunity via TGF-β2/TGFBR2 axis

**DOI:** 10.1186/s12916-019-1439-x

**Published:** 2019-11-15

**Authors:** Shu-zhen Zhang, Qin-qin Wang, Qiao-qiao Yang, Huan-yu Gu, Yan-qing Yin, Yan-dong Li, Jin-can Hou, Rong Chen, Qing-qing Sun, Ying-feng Sun, Gang Hu, Jia-wei Zhou

**Affiliations:** 1 Institute of Neuroscience, State Key Laboratory of Neuroscience, CAS Center for Excellence in Brain Science and Intelligence Technology, Shanghai Institutes for Biological Sciences, Chinese Academy of Sciences, 320 Yueyang Road, Shanghai, 200031 China; 2 School of Future Technology, University of Chinese Academy of Sciences, Beijing, 100049 China; 3 Neurobiology Key Laboratory, Jining Medical University, Jining, 272067 Shandong China; 4 Jiangsu Key Laboratory of Neurodegeneration, Department of Pharmacology, Nanjing Medical University, Nanjing, 210029 Jiangsu China; 5 Center for Brain Disorders Research, Center of Parkinson’s Disease, Beijing Institute for Brain Disorders, Capital Medical University, Beijing, 100053 China; 6 Co-innovation Center of Neuroregeneration, School of Medicine, Nantong University, Nantong, 226001 Jiangsu China; 7 Shanghai Center for Brain Science and Brain-Inspired Intelligence Technology, Shanghai, 201210 China

**Keywords:** NG2 glia, Neuroinflammation, Microglia, TGF-β, CX3CR1, Parkinson’s disease

## Abstract

**Background:**

Brain innate immunity is vital for maintaining normal brain functions. Immune homeostatic imbalances play pivotal roles in the pathogenesis of neurological diseases including Parkinson’s disease (PD). However, the molecular and cellular mechanisms underlying the regulation of brain innate immunity and their significance in PD pathogenesis are still largely unknown.

**Methods:**

Cre-inducible diphtheria toxin receptor (iDTR) and diphtheria toxin-mediated cell ablation was performed to investigate the impact of neuron-glial antigen 2 (NG2) glia on the brain innate immunity. RNA sequencing analysis was carried out to identify differentially expressed genes in mouse brain with ablated NG2 glia and lipopolysaccharide (LPS) challenge. Neurotoxin 1-methyl-4-phenyl-1,2,3,6-tetrahydropyridine (MPTP)-treated mice were used to evaluate neuroinflammatory response in the presence or absence of NG2 glia. The survival of dopaminergic neurons or glial cell activation was evaluated by immunohistochemistry. Co-cultures of NG2 glia and microglia were used to examine the influence of NG2 glia to microglial activation.

**Results:**

We show that NG2 glia are required for the maintenance of immune homeostasis in the brain via transforming growth factor-β2 (TGF-β2)-TGF-β type II receptor (TGFBR2)-CX3C chemokine receptor 1 (CX3CR1) signaling, which suppresses the activation of microglia. We demonstrate that mice with ablated NG2 glia display a profound downregulation of the expression of microglia-specific signature genes and remarkable inflammatory response in the brain following exposure to endotoxin lipopolysaccharides. Gain- or loss-of-function studies show that NG2 glia-derived TGF-β2 and its receptor TGFBR2 in microglia are key regulators of the CX3CR1-modulated immune response. Furthermore, deficiency of NG2 glia contributes to neuroinflammation and nigral dopaminergic neuron loss in MPTP-induced mouse PD model.

**Conclusions:**

These findings suggest that NG2 glia play a critical role in modulation of neuroinflammation and provide a compelling rationale for the development of new therapeutics for neurological disorders.

## Background

Immune homeostasis in the central nervous system (CNS) is vital for normal neural functionality. As in peripheral organs and tissues, neuroinflammation in the CNS is one of the most important protective mechanisms used by the organism to eliminate noxious stimuli such as pathogens and damaged tissues [1, 2]. An excessive inflammatory process unavoidably causes normal tissue damages and functional compromises for the host [3]. Normally, this damage is suppressed by integrated regulatory responses that promote the initiation of healing*.* Uncontrolled neuroinflammation is crucial for the pathogenesis of neurodegenerative diseases and mental disorders [4–6], indicating the importance of maintaining CNS functionality through immune homeostasis that is dependent on the delicate balance between pro-inflammatory and anti-inflammatory factors. In the peripheral tissues, the progression of acute inflammation is tightly controlled and the resolution program is quickly launched by the reactions of monocytes and inflammatory neutrophils once the pathogens or tissue debris are cleared [7]. Advances in understanding the cellular mechanisms underlying the resolution of inflammation in the peripheral system are paving the way for the development of anti-inflammatory drugs [8]. However, in the adult CNS, regulation of the resolution of inflammation remains elusive. Thus, an understanding of the molecular and cellular mechanisms underlying the resolution of neuroinflammation is critical for advancing our understanding of brain immune homeostasis and the associated brain diseases.

Accumulating evidence has indicated that the delicate balance of immune homeostasis in the CNS is dependent on complex cross-talk between diverse groups of cells in the brain, such as neuron–microglial and astrocyte–microglial interactions which play pivotal roles in constitutively keeping microglia in their resting state. Neuronal cells are very important modulators of inflammatory responses in the CNS [9, 10]. Neurons and microglia interact with each other through multiple pathways including CX3CL1-CX3CR1 axis, in which CX3CL1, a neuron-associated chemokine, modulates microglia-induced neurotoxicity by activating its receptor CX3CR1 that is primarily localized in microglia in the CNS [11]. CX3CR1 deficiency dysregulates microglial responses and causes more extensive neuronal cell loss, resulting in neurotoxicity in a toxic model of Parkinson’s disease (PD) and a transgenic model of amyotrophic lateral sclerosis [12]. In agreement with these findings, CX3CL1-mediated activation of CX3CR1 signaling reduces neurotoxicity and microglial activation in a rat model of PD [13, 14].

Moreover, neuronal cells also control microglia activity by producing “off” signals, such as CD200 and CD47, to maintain microglia in a quiescent homeostatic state and to antagonize pro-inflammatory activity. However, under pathological conditions, activated astrocytes produce “on” signals including chemokines and iNOS, facilitating microglia activation [5]. Thus, both microglia and astrocytes become over-activated and detrimental leading to severe neuroinflammation that contributes to neuronal damage. How the brain restrains this inflammation and whether an endogenous cell population(s), functioning as an immunosuppressor, exists in the CNS during the inflammatory response remain elusive.

NG2 glia are one of the four large glial cell populations in the CNS in addition to astrocytes, microglia, and oligodendrocytes [15]. Emerging evidence suggests that NG2 glia not only function as precursors of myelinating oligodendrocytes during development for the generation of oligodendrocytes which produce myelin sheaths around axons, but also play a role in other physiological processes, such as body weight control, cognition, and regulation of the immune response [16–19]. NG2 glia in the adult brain are known to have the capacity to proliferate and to differentiate into mature and myelinating oligodendrocytes throughout lifetime. Notably, the large majority of NG2 glia in the adult brain is maintained in a quiescent state under physiological conditions [20], although all NG2+ cells are endowed with the capacity to divide. NG2 glia responds to various CNS injuries and participates in glial scar formation by proliferation and accumulation in the injury sites [21]. NG2 glia also contribute to remyelination after insult [22].

Interestingly, uniform distribution of NG2 glia in the adult CNS including those regions with few oligodendrocytes [23], such as the substantia nigra (SN), indicates that they may play additional roles rather than just being oligodendrocyte precursors. Moreover, a wide range of immunomodulatory molecules, including various cytokines, chemokines, complement, and complement receptor molecules, have been shown to be expressed on NG2 glia [18, 19]. Further, it has been demonstrated that in postmortem brain specimens from patients with Alzheimer’s disease, there is a reduction in NG2 immunoreactivity which is negatively correlated to microglial immunoreactivity [24]. These data imply a potential role for NG2 glia in modulating neuroinflammation [25]. Recently, a study by Nakano et al. demonstrated that NG2 glial cells maintain neuronal function and survival in the hippocampus via the control of neuroimmunological function [26]. However, the role of NG2 glia in brain innate immunity and the underlying molecular and cellular mechanisms remain largely unknown.

In this study, we investigated the role of NG2 glia in the regulation of neuroinflammation and identified NG2 glia as potent immunosuppressors of the brain innate immunity. We demonstrated that microglia in mice with ablated NG2 glia showed aberrant activation and contributed the lipopolysaccharide (LPS)-induced neuroinflammatory response. We showed that NG2 glia-derived transforming growth factor-β2 (TGF-β2) signaling is critical for NG2 glia-mediated control of microglial activation. We also found that ablation of NG2 glia exacerbated dopaminergic neuronal cell loss in mouse PD model. Our results indicate that NG2 glia are vital negative regulators of neuroinflammation in the adult mouse brain.

## Methods

### Human tissue collection

Fresh-frozen ventral mesencephalic tissues of three pairs of age- and gender-matched PD patients and healthy controls were obtained from the Netherlands Brain Bank, Netherlands Institute for Neuroscience, Amsterdam, the Netherlands [27]. All the materials have been collected from donors for or from whom a written informed consent for a brain autopsy and the use of the material and clinical information for research purposes had been obtained by the Netherlands Brain Bank. All the PD subjects were clinically and neuropathologically diagnosed, and the healthy control subjects were devoid of any neurologic diseases.

### Animals

Adult (2~6 months old) C57BL/6 mice or neonatal Sprague-Dawley rat were from Shanghai Laboratory Animal Center, Chinese Academy of Sciences. The transgenic mouse strains *Tg (Cspg4-cre)1Akik/J (NG2-Cre)*, *Gt (ROSA)26Sor*^*tm1(HBEGF)Awai*^*/J*, *B6.129P2(Cg)-Cx3cr1*^*tm1Litt*^*/J* and *B6.Cg-Tg (Plp1-cre/ERT)3Pop/J* were purchased from the Jackson Laboratory (USA). *Olig1-Cre* transgenic mice in a C57BL/6 genetic background were generous gifts from Dr. Richard Lu [28]. Those strains that were originally not in a C57BL/6 (inbred) genetic background were back-crossed for ten generations prior to use. Characterization and genotyping of these mice were described previously [29]. They were maintained on a 12-h light/dark cycle at 23 °C with food and water available ad libitum.

### Tamoxifen treatments

Tamoxifen (TAM; Sigma-Aldrich, T5648) was made freshly by dissolving in 95% corn oil (Sigma-Aldrich)/5% ethanol solution at room temperature with intermittent vortexing. The final concentration of TAM was 20 mg ml^−1^. Mice were injected intraperitoneally with 80–100 mg kg^−1^ daily for 5~8 consecutive days.

### Cell ablation with diphtheria toxin

Cell-type-specific Cre mice were crossed to generate with inducible diphtheria toxin receptor (iDTR) mice to allow expression of DTR in the targeted cell populations. The DTR is expressed after Cre recombinase removes the STOP cassette, increasing susceptibility of specific cell type to diphtheria toxin (DT; Sigma-Aldrich, D0564). Their littermates bearing Cre recombinase were also administrated with DT and used as control. No specific adverse side effects of DT were observed when administered to the control and iDTR mice.

For NG2 glia ablation paradigm, DT administration was performed as described previously with some modifications [30]. In brief, adult mice (2~6 months old) were administrated with seven intraperitoneal injections of DT (150 ng/mouse) at 12-h intervals (i.e., for 3.5 consecutive days in total). This administration paradigm ensures efficient and specific ablation of NG2 glial cells, given that depletion of NG2 glia is usually followed by a fast repopulation of the cells [20, 31]. Twelve hours after the final DT injection, mice received single intraperitoneal injection of LPS (2.5 mg kg^−1^, Sigma-Aldrich, L2630). Animals were sacrificed 4 h later.

### Sample collection and preparation for RNA-seq analysis

Briefly, the striatum of 2~3-month-old *DTR*^*NG2*^ Tg mice and littermate control (NG2-Cre) were homogenized in TRIzol reagent (Invitrogen, Carlsbad, California, USA) 4 h after LPS challenge. A total amount of 3 μg RNA per sample was used as input material for the RNA sample preparations. Sequencing libraries were generated using NEB Next® Ultra RNA Library Prep Kit for Illumina® (NEB, USA) following the manufacturer’s recommendations, and index codes were added to attribute sequences to each sample.

Briefly, mRNA was purified from total RNA using poly-T oligo-attached magnetic beads. Fragmentation was carried out using divalent cations under elevated temperature in NEBNext First Strand Synthesis Reaction Buffer (5X). First-strand cDNA was synthesized using random hexamer primer and M-MuLV Leading Edge Genomic Services & Solutions Reverse Transcriptase (RNase H-). Second-strand cDNA synthesis was subsequently performed using DNA polymerase I and RNase H. Remaining overhangs were converted into blunt ends via exonuclease/polymerase activities. After adenylation of 3′ ends of DNA fragments, NEBNext Adaptor with hairpin loop structure was ligated to prepare for hybridization. In order to select cDNA fragments of preferentially 250~300 bp in length, the library fragments were purified with AMPure XP system (Beckman Coulter, Beverly, USA). Then 3 μl USER Enzyme (NEB, USA) was used with size-selected, adaptor-ligated cDNA at 37 °C for 15 min followed by 5 min at 95 °C before PCR. Then PCR was performed with Phusion High-Fidelity DNA polymerase, Universal PCR primers and Index (X) Primer. At last, PCR products were purified (AMPure XP system) and library quality was assessed on the Agilent Bioanalyzer 2100 system.

### Clustering and sequencing

The clustering of the index-coded samples was performed on a cBot Cluster Generation System using TruSeq PE Cluster Kit v3-cBot-HS (Illumina) according to the manufacturer’s instructions. After cluster generation, the library preparations were sequenced on an Illumina Hiseq platform and 125-bp/150-bp paired-end reads were generated.

### Quality control

Raw data (raw reads) of fastq format were firstly processed through in-house perl scripts. In this step, clean data (clean reads) were obtained by removing reads containing adapter, reads containing ploy-N, and low-quality reads from raw data. At the same time, Q20, Q30, and GC content of the clean data were calculated. All the downstream analyses were based on the clean data with high quality.

### Reads mapping to the reference genome

Reference genome and gene model annotation files were downloaded from genome website directly. Index of the reference genome was built using Hisat2 v2.0.5, and paired-end clean reads were aligned to the reference genome using Hisat2 v2.0.5.

### Quantification of gene expression levels

Feature Counts v1.5.0-p3 was used to count the reads numbers mapped to each gene. And then FPKM of each gene was calculated based on the length of the gene and reads count mapped to this gene.

### Differential expression analysis

Differential expression analysis of two conditions was performed using the edgeR R package (3.18.1). The *P* values were adjusted using the Benjamini and Hochberg method. Corrected *P* value of 0.05 and absolute fold change of 2 were set as the threshold for significantly differential expression.

### KEGG enrichment analysis of differentially expressed genes

KEGG is a database resource for understanding high-level functions and utilities of the biological system (http://www.genome.jp/kegg/). We used cluster Profiler R package to test the statistical enrichment of differential expression genes in KEGG pathways.

### Western blot analysis and quantification

Western blotting was performed as described previously [32]. The following primary antibodies were used: (a) rabbit anti-IL-1β pAb (1:1000; Abcam, ab9722), (b) mouse anti-β-actin mAb (1:5000; Sigma-Aldrich, A5441), (c) rabbit anti-phosphor-SMAD2 pAb (1:1000; Invitrogen #51-1300), (d) rabbit anti-SMAD2 pAb (1:250; Cell signaling #3108), (e) rabbit anti-TNF-α pAb (1:100; Abcam, ab9739), and (f) mouse anti-Olig1 mAb (1:1000; Millipore, mab5540). The membrane was washed and incubated for 1 h at room temperature with the corresponding secondary antibodies: (a) HRP-conjugated goat anti-rabbit IgG (1:10,000; Jackson ImmunoResearch Laboratories, 115-035-003) and (b) HRP-conjugated goat anti-mouse IgG (1:10,000; Jackson ImmunoResearch Laboratories, 115-035-003). Peroxidase activity was detected with SuperSignal WestPico chemiluminescent substrate (Pierce Biotechnology) and visualized and digitized with ImageQuant (LAS-4000, Fujifilm, Japan). Optical densities of bands were analyzed by using ImageJ software (NIH, USA). Protein levels, quantified by computer analysis as the ratio between each immunoreactive band and the levels of β-actin, were expressed as a percentage of vehicle-treated control.

### Immunofluorescence, confocal microscopy, and image analysis

Sections or fixed cell cultures were incubated with one primary antibody followed by incubation with secondary antibody conjugated with either Alex488 or Alex555. The same sections were then incubated with another primary antibody, followed by incubation with the appropriate secondary antibody. Sections were imaged using either a cooled CCD (DP72, Olympus) on a microscope (BX51; Olympus) or a laser confocal microscope (Leica). Data were obtained and processed using Fiji or Adobe Photoshop 7.0 software (Adobe Systems). In some cases, immunosignals were visualized by using 3,3-diaminobenzidine (Sigma-Aldrich).

The following primary antibodies were used: (a) rabbit anti-NG2 pAb (1:500, a gift of W Stallcup), (b) guinea pig anti-NG2 pAb (1:500, a gift of W Stallcup), (c) rabbit anti-NG2 pAb (1:500, Millipore, ab5320), (d) mouse anti-CC1 mAb (1:200, Calbiochem, OP80), (e) guinea pig anti-NeuN pAb (1:1000, Millipore, ABN90), (f) rabbit anti-IBA1 pAb (1:500; WAKO, 019-19741), (g) mouse anti-TGF-β1 mAb (1:500, R&D System, mab240), (h) rabbit anti-TGF-β2 pAb (1:500, Santa Cruz, sc-90), (i) rabbit anti-GFAP pAb (1:1000; DAKO, Z0334), (j) mouse anti-GFAP mAb (1:500, Sigma-Aldrich, G3893), (k) rabbit anti-lectin (1:1000; Sigma, L0401), and (l) mouse anti-MBP mAb (1:500, Calbiochem, ne1019).

### Cell counting

The number of tyrosine hydroxylase-positive cells was quantified in brain cryosections with typical morphology of the substantia nigra, as described previously [5]. The number of NG2+, NeuN+, CC1+, or IBA+ cells was quantified in at least three sections from four serial brain sections per animal using a similar approach.

### Intensity analysis

Average intensities of TGF-β2 immunoreactivity were calculated as described previously [5]. Briefly, using software ImageJ (NIH, USA), sampling was conducted in a 28 × 28 pixel area, in the indicated brain regions, in 40 images taken from 4 to 8 consecutive sections. Values are reported as average intensity above background ± SEM.

### Mouse stroke model

Mouse stroke model was prepared according to the protocol described previously [33] with a few modification. Briefly, mice were deeply anesthetized with pentobarbital sodium (30 mg kg^−1^, i.p). The left common carotid artery was sutured to block the supply of blood to the brain for 4 h. The reperfusion was performed through the clearance of the suture from the common carotid artery of lumen 4 h following the occlusion. The body temperature of the mice was kept at 37 °C during surgery.

### Primary NG2 glial cell cultures

*NG2 glial cells* were prepared from the cerebrocortex and hippocampus of Sprague Dawley rats at P0, as described previously [34] with a few modifications. Briefly, the neonatal brains were trypsinized and dissociated and cells were plated at density of 1~2 × 10^6^ cells/75-cm^2^ flask (Corning) without poly-l-lysine (PLL) pre-treatment in Dulbecco’s modified Eagle medium (DMEM) containing 10% FBS, 1% penicillin/streptomycin, and 1% glutamine for 40 min at 37 °C. The suspended cells were then transferred to the PLL-coated flasks. Culture media were changed every 3 days. Between 6 and 8 days in vitro, culture flasks (with cell mixtures) were shaken on a horizontal orbital shaker (250 rpm) at 37 °C for 1 h (or 120 rpm for 2 h) to remove microglia cells, followed by shaking at 250 rpm for 16 h for the collection of NG2 glial cells. The medium with NG2 cells was centrifuged at 900 rpm for 3 min. The pellets were resuspended in DMEM containing 10% FBS, 1% penicillin/streptomycin, and 1% glutamine. And NG2 glial cells were seeded to a 6-well plate at a density of 1.2 × 10^6^ cells per well. Four hours after plating, the medium was changed to DMEM/Ham’s F12 medium containing 5 ng ml^−1^ FGF-2 (Sigma-Aldrich) and 10 ng^−1^ PDGF-AA (Peprotech), 1% penicillin/streptomycin, and 1% glutamine. Cells were maintained at 37 °C in 95% air-5% CO_2_ humidified atmosphere. The purity (80~90%) of these cultures was confirmed by NG2 immunocytochemistry. NG2 glial cell-conditioned medium was collected 48 h later and was filtered through a 0.22-μm filter unit (Millipore) before use.

### Primary microglial cultures

Microglial cultures were prepared as described previously [5]. In brief, neonatal rats, age 1–3 days, were used for the microglia isolation. Microglial cultures were maintained in DMEM/Ham’s F12 complete medium containing 10% heat-inactivated FBS, 1% penicillin and streptomycin until use. The purity (> 99%) of these cultures was confirmed by IBA1 immunocytochemistry.

### Co-culture of NG2 glia with microglia

We adopted two different co-culture systems. In case of mixed co-cultures, rat microglial cells were seeded onto PLL (0.1 mg ml^−1^, Sigma-Aldrich)-coated plates at a concentration of 1.5 × 10^4^ cells/cm^2^. Twelve hours after plating, rat NG2 glial cells were added directly into the microglial culture allowing cell–cell contact at various ratios. The co-cultures were maintained in the culture medium containing DMEM and 10% FBS for 2 h. The medium was then switched to the conditioned medium of NG2 glial cells (100%) supplemented with 0.5% FBS. On day 3 in vitro, the cells were treated with 50 ng ml^−1^ LPS or saline for 4 h prior to harvest for qPCR or western blot analysis.

An alternative co-culture system of microglial cells with NG2 glial cells was also used. Briefly, freshly prepared rat primary microglial cells and NG2 glial cells were seeded onto PLL-coated coverslips at a density of 2 × 10^4^ cells/cm^2^ and maintained in culture medium as described above. Meanwhile, rat primary NG2 glial cells were also seeded onto separate PLL-coated coverslips at the same density and were maintained in 100% conditioned medium of NG2 glial cells supplemented with 0.5% FBS. The two types of cells were cultured independently. Forty-eight hours after incubation, the coverslips in which NG2 glial and microglial cells are set, respectively, were transferred to new culture dishes with the conditioned medium of NG2 glial cells (100%) and placed in a side-by-side manner at a ratio of 1:1. The co-cultures were exposed to LPS (50 ng ml^−1^) for 4 h prior to harvest for further analysis.

### Quantitative assessment of microglial morphology

After immunohistochemistry and image acquisition, the images were imported into ImageJ (NIH, USA) for the morphological measurement of microglia, as previously described [35]. Average cell diameter, endpoints, and process length of microglia in the cerebrocortex region were calculated from six brain sections for one animal. Three to five animals per group were used to generate the statistical data.

### Enzyme-linked immunosorbent assay

Conditioned medium was collected from primary cultured rat NG2 glial cells and centrifuged at 2000*g* for 10 min at 4 °C to remove cell debris. This was followed by centrifugation at 5000*g* for 20 min using Centricon® centrifugal filter units with a molecular weight cutoff of 3 kDa. The supernatant was used for measurements of TGF-β2. The levels were quantified by using ELISA Kit (Invitrogen, MA; Eagle Biosciences, Inc., NH, USA) according to the instructions of the manufacturer.

### Isolation of adult primary microglia

Mouse brain microglia were isolated from the cerebrocortex of *DTR*^*NG2*^ Tg and their littermate control using MACS Technology (Miltenyi Biotec) according to the manufacturer’s instructions. Briefly, brain tissues were dissected using the Neural Tissue Dissociation Kit P (Miltenyi Biotec, 130-092-628). CD11b-positive microglia were magnetically labeled with CD11b MicroBeads (Miltenyi Biotec, 130-093-634), loaded onto a MACS Column and subjected to magnetic separation. Isolated cells were homogenized in TRIzol reagent immediately for further analysis (Invitrogen, Carlsbad, CA, USA).

### TGFBR2 knockdown

Knocking down *TGFβR2* (GenBank accession number NM_009371.3) was performed in mouse microglial cell line BV2 cells (a gift of WD Le). The cell line shows some characteristic features of microglia, such as hyper-responsiveness to LPS stimuli. No overt mycoplasma contamination was detected. The cells were seeded at a density of 2 × 10^6^ cells in a 6-well tissue culture dish 36 h before transfection with 2 nM small interfering RNA (siRNA) targeting *TGFβR2* or control siRNA (Jima, Shanghai, China) using electroporation (Amaxa Nucleofector®, Germany). siRNA duplexes used were as follows: scrambled: r (ACGUGACACGUUCGGAGAATT)dTdT; antisense #1: r (UCUUCCAUGUUACAGGCACTT)dTdG; antisense #2: r (UGUUAGAGCUCUUGAGGUCTT)dTdG; and antisense #3: r (AUUUCCCAGAGUACCAGAGTT)dTdG. Forty-eight hours after transfection, cells were harvested for qPCR analysis.

### Viral injection

Lentiviral vectors encoding human CX3CR1 (NM_001171171.1) and TGF-βR2 (NM_003242.6) were prepared by Shanghai OBIO company. The titers of the vectors in this study were 3 × 10^8^ TU/ml. Mice were deeply anesthetized with pentobarbital sodium (30 mg kg^−1^, i.p) and placed in a stereotaxic frame. A total of 1.8 × 10^6^ TU of lentivirus (Lv.CMV.GFP or Lv.CMV.cx3cr1.GFP or Lv.CMV.TGF-βR2.GFP.) was injected bilaterally into the mouse striatum using Hamilton syringe. The stereotaxic injection coordinates were AP + 0.5, ML ± 1.6 and 2.4, and DV − 4.5~ − 2.5 mm from the bregma. Mice were monitored daily for 7 to 11 days according to experimental demand.

### RNA isolation and quantitative PCR

RNA isolation and quantitative PCR were performed as described previously [5]. Briefly, quantitative PCR was performed with SYBR-Green premix Ex Taq (Takara, Japan) and detected by a Real Time PCR System (Roche LightCycler 480 or Rotorgene 6000). β-Actin was used as an internal control gene. qPCR primers were designed using Primer Picking Program. The primer sequences for amplifying mouse cDNA fragments were as follows: *β-actin*, forward, 5′-CGTCGACAACGGCTCCGGCATG-3′, reverse, 5′-CACCATCACACCCTGGTGCCTAGG-3′; *CX3CR1*, forward, 5′-TTCCCATCTGCTCAGGACCTC-3′, reverse, 5′-CAGACCGAACGTGAAGACGA-3; *IBA1*, forward, 5′-GGAGATTTCAAAAGCTGATGTGGA-3′, reverse, 5′-CCTCAGACGCTGGTTGTCTT′-3′; *iNOS*, forward, 5′-TGCTTTGTGCGAAGTGTCAG-3′, reverse, 5′-CCCTTTGTGCTGGGAGTCAT-3′; *IL-1β*, forward, 5′-AGGAGAACCAAGCAACGACA-3′, reverse, 5′-CTTGGGATCCACACTCTCCAG-3′; *IL-6*, forward, 5′-GCCTTCTTGGGACTGATGCT-3′, reverse, 5′-TGCCATTGCACAACTCTTTTCT-3′; *IL-12β*, forward, 5′-TGGTTTGCCATCGTTTTGCTG-3′, reverse, 5′-ACAGGTGAGGTTCACTGTTTCT-3′; *NeuN*, forward, 5′-CCACCACTCTCTTGTCCGTT-3′, reverse, 5′-ATCAGCAGCGGCATAGACTC-3′; *NG2*, forward, 5′-GGCTTGTGCTGTTCTCACA-3′, reverse, 5′-CACAGACTCTGGACAGACGG-3′; *Olig1*, forward, 5′-CTCGCCCAGGTGTTTTGTTG-3′, forward, 5′-TATAAGCCTGCGCTACGACG-3′; *Pdgfrα*, forward, 5′-TGGCAAAGAACAACCTCAG-3′, reverse, 5′-CGATAACCCTCCAGCGAAT-3′; *Pdgfrβ*, forward, 5′-CATGTCTGAGACCCGGTACG-3′, reverse, 5′-CTCTGCAGGTAGACCAGGTG-3′; *PLP1*, forward, 5′-AGCGGGTGTGTCATTGTTTG-3′, reverse, 5′-GCGCAGAGACTGCCTATACT-3′; *TNF-α*, forward, 5′-ACGTCGTAGCAAACCACCAA-3′, reverse, 5′-ATAGCAAATCGGCTGACGGT-3′; *Csf1r*, forward, 5′-CTCTTCCTCTGTTCCCTTTCAGG-3′, reverse, 5′-AGTTCTGTGAGGACGGGAAC-3′; *Sall1*, forward, 5′-AACTAAGCCGAGGACCAAGC-3′, reverse, 5′-CTTCGGGGTCGGATTGGAAA-3′; *Trem2*, forward, 5′-GGGTCACCTCTAGCCTACCA-3′, reverse, 5′-GTACCTCCGGGTCCAGTGA-3′; *Tmem119*, forward, 5′-CACCCAGAGCTGGTTCCATA-3′, reverse, 5′-GTGACACAGAGTAGGCCACC-3′; *p2ry12*, forward, 5′-ACCACCCCTGTTTTTCCAGT-3′, reverse, 5′-AGGCAGCCTTGAGTGTTTCTG-3′; *p2ry13*, forward, 5′-TGGGTTGAGCTAGTAACTGCC-3′, reverse, 5′-TTGTCCCGAGCATCAGCTTT-3′; *MBP*, 5′-CACCACTCTTGAACACCCCA-3′, reverse, 5′-TGCCCACGCTTCTCTTCTTT-3′; and *ALDH1L1*, 5′-CCCGTCTTTGACCTTGGGTG-3′, reverse, 5′-GAACTTAAACACGGGCACGC-3′. The primer sequences for amplifying rat cDNA fragments were as follows: β-actin, forward, 5′-ACCCGCCACCAGTTCGCCAT-3′, reverse, 5′-CTAGGGCGGCCCACGATGGA-3′; CX3CR1, forward, 5′-GAGTGGCTGGCACTTCCTG-3′, reverse, 5′-CACCAGACCGAACGTGAAGA-3′; and *IL-1β*, forward, 5′-TGCAGCTGGAGAGTGTGGATCC-3′, reverse, 5′-ACCAGTTGGGGAACTGTGCAGA-3′. The primer sequences for amplifying human cDNA fragments were as follows: *TGFβR2*, forward, 5′-CACTGACAACAACGGTGCAGTC-3′, reverse, 5′-GCTTGGGGTCATGGCAAACTG-3′; and *CX3CR1*, 5′-AGGTGGCCAAACACTGAGAC-3′, reverse, 5′-GTGAAGGCCTCTAGTCGCTG-3′. Following PCR amplification, a first derivative melting-curve analysis was performed to confirm the specificity of the PCR. The relative fold difference in mRNA between samples was calculated by comparing the threshold cycle (*C*_t_) at which product initially appeared above background according to 2^−(∆ *C*t)^, where *∆C*_t_ is the difference between the control group and a treatment group.

### Evans blue assay

The Evans blue assay was performed according to previous studies [36, 37]. Briefly, after 3.5 days of DT injection, NG2-Cre/iDTR and NG2-Cre mice were injected with 100 μl 2% solution of Evans blue in normal saline (4 ml kg^−1^ of body weight) intraperitoneally. Four hours after the dye injection, the mice were transcardially perfused with 50 ml of ice-cold PBS, and the brain tissue was collected and divided into right and left hemispheres. The samples were homogenized in 1100 μl of PBS, sonicated and centrifuged (30 min, 14,000*g*, 4 °C). The supernatant was collected in aliquots. To each 500 μl aliquot, an equal amount of 50% trichloroacetic acid was added, incubated overnight at 4 °C, and centrifuged (30 min, 14,000*g*, 4 °C). Evans blue stain was measured by a spectrophotometer at 610 nm and quantified according to a standard curve.

### MPTP-induced mouse PD model

Mice were administered with neurotoxin l-methyl-4-phenyl-l,2,3,6-tetrahydropypridine (MPTP; 20 mg kg^−1^, i.p.) for four times at 2-h intervals as described previously [38, 39], and the total dosage per mouse was 80 mg kg^−1^. The animals were perfused with 4% paraformaldehyde in 0.1 M PB (pH 7.4) at day 7 post MPTP administration, and coronal cryosections at a thickness of 25 μm were prepared for immunohistochemistry.

### Statistical analysis

Statistical analysis was performed using GraphPad software (GraphPad Prism v7.0; GraphPad Software). Data presented as mean ± SEM or individual values and mean. They were submitted to nonparametric test with Mann–Whitney test or two-tailed *t* test or one-/two-way ANOVA followed by Bonferroni’s multiple comparisons test (as a post hoc test). *P* < 0.05 was considered as significant in statistics.

## Results

### NG2 glial cell ablation exacerbates neuroinflammation following LPS treatment

**Fig. 1:**
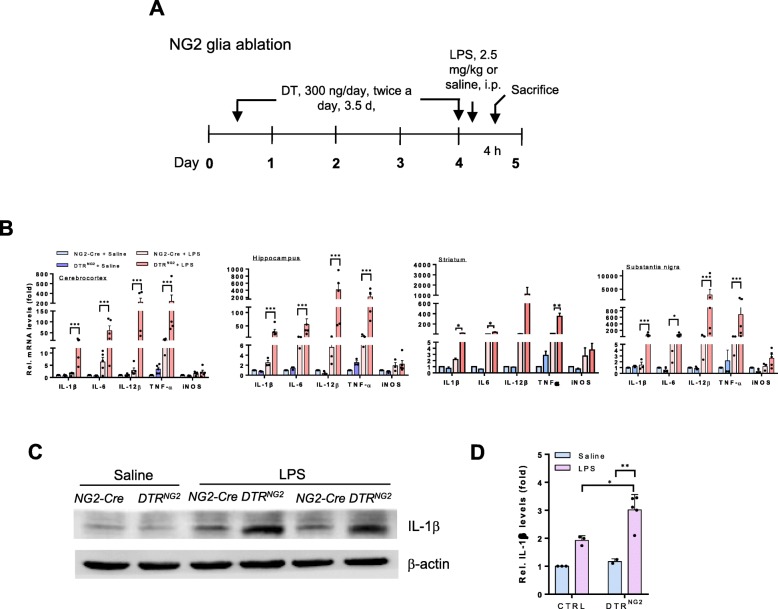
Ablation of NG2 glia exacerbates neuroinflammation in response to LPS. **a** Schematic representation of the administration paradigm used for NG2 glia ablation in iDTR adult mouse brain using diphtheria toxin (DT) administration (i.p.). DT (300 ng/day) was given twice a day for 3.5 days. **b** Representative graph showing relative mRNA levels of inflammatory mediators in the indicated brain regions from *DTR*^*NG2*^ and control mice. **c** The expression of IL-1β in the striatum of *DTR*^*NG2*^ Tg mouse brain. Immunoblot analyses of tissue lysates from *DTR*^*NG2*^ Tg mice and their counterparts 4 h after LPS challenge. **d** Quantification of data shown in **c**. Error bars represent mean ± SEM; (*n* = 2–5). Two-way ANOVA with Bonferroni’s post test was used. *P<0.05, **P < 0.01, ***P < 0.001

Previous studies have shown that LPS administration via either systemic or direct injection into the brain causes robust neuroinflammation [2, 40, 41]. We thus used a mouse model of systemic LPS administration (single dose, 2.5 mg kg^−1^, i.p.) for investigating the potential role of NG2 glia in the neuroinflammation. Following LPS challenge, microglia are one of the major sources for pro-inflammatory mediator production and NG2 glia are activated in response to brain injuries [42]. To investigate the potential function of NG2 glia in neuroinflammation directly in vivo, we generated a double-transgenic (Tg) mouse line in which NG2 glia can be ablated in a temporally controlled manner. *NG2-Cre* Tg mice were crossed with Cre-inducible diphtheria toxin receptor (iDTR) Tg mice [30] to generate conditional *DTR* Tg mice, in which DTR was preferentially expressed in NG2 glia. In this mouse line, DTR is only expressed after Cre recombinase deletes the STOP cassette, making only NG2-expressing cells susceptible to diphtheria toxin (DT). Following 3.5-day DT administration (Fig. 1a), as determined by immunostaining for NG2, ~ 50% of NG2 glial cells were ablated in multiple brain regions, including the cerebrocortex, corpus callosum, and striatum, compared to *NG2-Cre* transgenic littermates administered DT (Additional file 1: Figure S1).

Strikingly, a single injection of LPS caused dramatic increases (ranging from a few dozen to several-hundred-fold depending on the brain region and the individual molecules) in mRNA expression levels of pro-inflammatory mediators, including tumor necrosis factor-α (TNF-α) and interleukin (IL)-1β, IL-6, and IL-12β in the major brain regions of NG2 glia-ablated mice, compared with those in the non-ablated animals exposed to LPS (Fig. 1b). Consistent with these results, western blotting analysis showed a rapid increase in IL-1β protein levels (~ 3 fold) in the NG2 glia-ablated mouse brains after exposure to LPS compared to the brain of LPS-treated control animals (Fig. 1c,d), indicating that disruption of the NG2 glia network renders mice more sensitive to LPS. Of note, under basal conditions, except for TNF-α that showed a moderate increase in the NG2 glia-ablated brains compared with those of the controls, the expression of most of the pro-inflammatory mediators in the mouse brain after partial ablation of NG2 glia were comparable to those of the controls (non-ablation) (Fig. 1b), indicating that acute ablation of NG2 glia per se mildly perturbed brain innate immunity.

We examined whether the NG2 glia ablation was cell-type specific. Given that in addition to NG2 glia, NG2 gene is also expressed in pericytes, a constitutive element of the blood–brain barrier (BBB) [43], we determined whether the aberrant inflammatory response observed in brains with acute ablation of NG2 glia resulted from BBB leakage. We found that the permeability of the BBB was not profoundly altered in these animals, as evidenced by no marked Evans blue or IgG extravasation in the brain parenchyma after the ablation of NG2 glia compared to that observed in the control (Additional file 1: Figure S2a-c). The vascular network in the brain with NG2 glia ablation appeared to be highly similar to that of the control, as revealed by lectin staining of the vascular endothelium (Additional file 1: Figure S2c, d). The expression levels of PDGFRβ, a marker of pericytes, were not significantly altered between genotypes (Additional file 1: Figure S2e).

Furthermore, there was no overt inflammatory mononuclear cell infiltration in the NG2 glia-ablated mouse brain, as shown by levels of markers for monocytes not being significantly altered between genotypes, such as recombination-activating gene 1 (Rag1), lymphocyte-specific protein tyrosine kinase (Lck), interferon-γ (IFN-γ), and clonal stimulating factor (CSF) (Additional file 1: Figure S2f). The acute NG2 glial cell ablation strategy did not affect the survival of other major cell populations in the brain, including neuronal cells, astrocytes (Additional file 1: Figure S3), and microglia under basal conditions (Fig. 4a, b). Together, these data suggest that acute ablation of NG2 glial cells is achieved with a high cell-type specificity, and it is unlikely that acute NG2 glia ablation results in BBB leakage.

### Ablation of Olig1+ NG2 glia, but not mature oligodendrocytes, augments neuroinflammation following LPS exposure

To further verify whether the imbalance of cell signaling networks leading to the activation of inflammatory genes in *DTR*^*NG2*^ mouse brain following LPS stimulation truly results from NG2 glial cell ablation, we generated *Olig1-Cre/iDTR* (*DTR*^*Olig1*^) double-transgenic mice, because the transcription factor Olig1 is another marker of oligodendrocyte precursor cells [15, 44]. The efficacy of the ablation of NG2 glia in the DT-treated adult *DTR*^*Olig1*^ brains was up to 50% on average compared with that in the control brains (Additional file 1: Figure S4a-d). Similar to the *DTR*^*NG2*^ mice, no marked disruption of the BBB or cell loss of neurons, oligodendrocytes, astrocytes, or microglia was seen in the *DTR*^*Olig1*^ mice (Additional file 1: Figure S4e-h and Figure S7), suggesting that depletion of *Olig1*+ cells specifically targeted the NG2 glia.

**Fig. 2:**
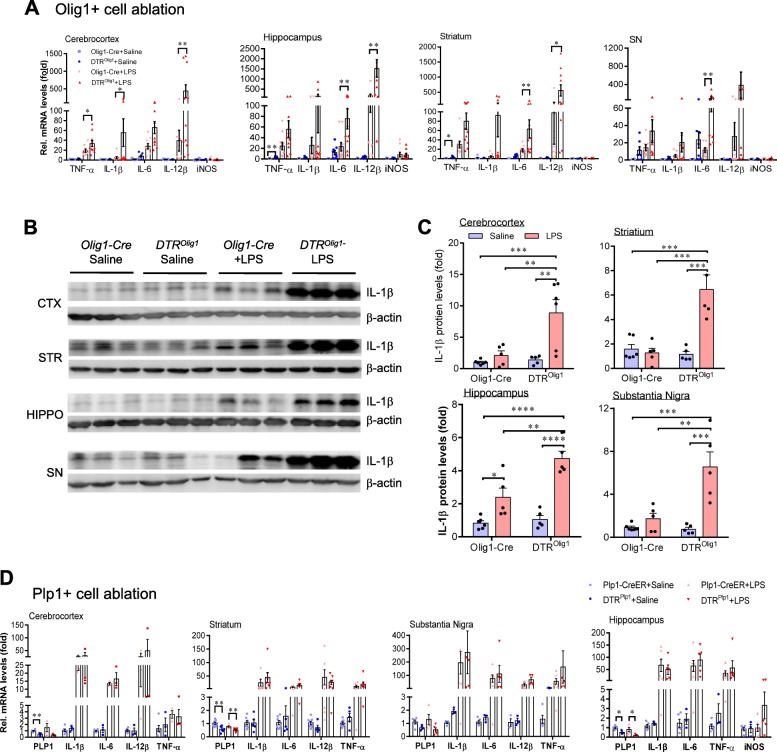
Olig1+ cell ablation augments neuroinflammation following LPS treatment. **a** Representative graph showing relative mRNA levels of inflammatory mediators in the indicated brain regions from *DTR*^*Olig1*^ Tg and littermate control animals (*n* = 9–13 mice). **b** Representative western blots showing IL-1β expression in the indicated brain regions from adult mice with Olig1+ cell ablation. *Olig1-Cre* serve as negative control. Animals were sacrificed 4 h after LPS challenge. **c** Quantification of data shown in **b** (*n* = 5–6 mice). **d** Oligodendrocyte ablation does not significantly alter the levels of inflammatory mediators in the adult mouse brain following LPS challenge. qPCR analysis of the indicated brain regions including the hippocampus, substantia nigra (SN), striatum, and cerebrocortex 4 h after systemic LPS (2.5 mg kg^−1^, i.p.) administration. Data are expressed as mean ± SEM for all the data. Two-way ANOVA with Bonferroni’s post test was used for the statistical analysis (*n* = 3–8). No significance is found. *P < 0.05, **P < 0.01, ***P < 0.001, ****P < 0.0001

Following systemic administration of LPS, these animals also exhibited remarkable elevations in the expression of gene transcripts encoding TNF-α, IL-1β, IL-6, and IL-12β, but not iNOS, in the brain (Fig. 2a). Consistent with these data, the levels of IL-1β protein were significantly increased in mice with ablated *Olig1*^+^ cells following LPS challenge compared with that of controls (Fig. 2b, c). Taken together, these data strongly suggest that NG2 glia are key negative regulators of neuroinflammation.

NG2 glia normally differentiate into oligodendrocytes during development [45]. We asked whether mature oligodendrocytes also contribute to the immune homeostasis in the brain. We crossed iDTR mice to myelin proteolipid protein 1 (*PLP1*)-CreERT2 Tg mice in which mature oligodendrocytes are partially ablated following repeated DT injection (Additional file 1: Figure S5). These mice displayed similar magnitude in the changes of pro-inflammatory mediators to the control animals either under the steady-state or in response to acute LPS stimulation (Fig. 2d), suggesting that myelin sheath damage does not evoke a marked inflammatory response, and mature oligodendrocytes have no profound contribution to the maintenance of the immune balance in the brain in response to acute LPS stimulation.

### NG2 glia control the transcriptional expression of inflammation-associated genes

**Fig. 3:**
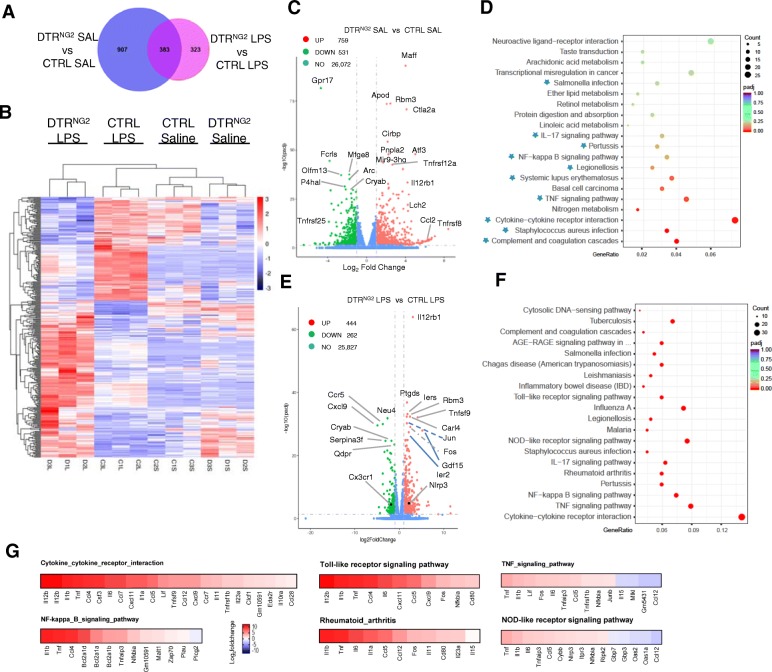
RNA sequencing analysis of the striatum reveals that NG2 glial cell ablation perturbs brain immune homeostasis and exacerbates neuroinflammation after exposure to LPS. **a** A Venn diagram analysis of the differentially expressed genes between *DTR*^*NG2*^ SAL and CTRL SAL mice or between *DTR*^*NG2*^ LPS and CTRL LPS mice. The sum of numbers shown in each big circle is the total number of differentially expressed genes in the comparison group. The overlapping part between the two circles is the number of differentially expressed genes that are common among the comparison groups. **b** Hierarchical clustering of genes regulated by the interaction of LPS treatment and NG2 cell ablation (i.e., the area in part of right circle in purple in **a**. Profiles are shown in a color scale where blue is low and red is high. *n* = 3 per group. **c** A volcano plot of *P* values as a function of weighted fold change between *DTR*^*NG2*^ saline and control groups. Red and green dots represent the upregulated and downregulated mRNAs. Some genes with higher significance values are annotated. **d** The top 20 differently regulated KEGG pathways by NG2 cell ablation alone. Asterisks indicate the 10 functional categories that are related to the immunological response. **e** A volcano plot of *P* values as a function of weighted fold change between *DTR*^*NG2*^ LPS and CTRL LPS groups. Some genes with higher significance values are annotated. **f** The top 20 differently regulated KEGG pathways between *DTR*^*NG2*^ LPS and CTRL LPS groups. **g** Heatmaps showing the expression (log_2_ ratio) of differentially expressed genes of six functional categories that show the most dramatic changes according to their indicated KEGG pathways

Next, we assessed global transcriptional changes in the NG2 glia-ablated mouse brain by comparing striatal gene transcript expression profiles between *DTR*^*NG2*^ Tg mice and control animals challenged with or without LPS using RNA sequencing (RNA-seq). Analysis of differentially expressed genes between NG2 glia ablation alone and control SAL showed 759 upregulated and 531 downregulated genes (with > 2-fold change and *P* < 0.05) among all the 27,362 detected genes (Fig. 3a–c). Hierarchical clustering showed a clear segregation of the genes that were differentially expressed between the NG2 glia-ablated brains and controls (Fig. 3b). The expression of GPR17, a NG2 glia-specific gene [46], was significantly downregulated in mice with ablated NG2 glia (Fig. 3c), indicating an efficient NG2 ablation. We observed pronounced increases in the levels of inflammation-associated genes, such as *Tnfrsf12a*, *Il12rb1*, *Maff* [47], *Ctla2a*, *Ccl2*, and *Arf3*, following NG2 glia ablation compared to the control. In contrast, there were remarkable decreases in the homeostatic microglia signature molecule *Olfm13* [48] and negative regulators of the immune response, such as *Gpr17* [49] and *Cryab* [5] in the brain tissues compared to the controls (Fig. 3c). These data suggest that NG2 glia ablation perturbs brain homeostasis by altering underlying gene transcriptional network.

To gain an insight into the mechanistic process of inflammation in the brain, we performed functional categorization using the bioinformatics database (http://www.genome.ad.jp/kegg/) for KEGG enrichment analysis of biological processes. As expected, 10 out of the 20 functional categories of the most significantly enriched genes observed to be altered following NG2 glia ablation were related to the immunological response (Fig. 3d and Additional file 1: Figure S6a). These observations are in accordance with the results showing an increase in the levels of TNF-α in mice lacking NG2 glia (Fig. 1b). Taken together, these data emphasize that the maintenance of brain innate immunity depends on the integrity of the NG2 glia network in vivo.

Furthermore, we performed gene expression analysis on mice with NG2 glia loss after LPS challenge. Among all the 26,533 detected genes, there were 444 upregulated and 262 downregulated genes between the *DTR*^*NG2*^ LPS and LPS alone mice. Hierarchical clustering showed a clear segregation of the genes that were differentially expressed between genotypes in response to LPS (Fig. 3b–e). KEGG enrichment analysis of biological processes showed that the NG2 glia-ablated brains became highly inflamed following LPS challenge, given that 17 out of the top 20 biological pathways most significantly enriched among the genes observed to be altered were related to inflammation and the immune response (Fig. 3f). Consistent with these data, the combination of NG2 glia ablation and LPS challenge significantly elevated the levels of pro-inflammatory mediators, which are associated with cytokine and cytokine receptor interactions, the toll-like receptor signaling pathway, the Tnf signaling pathway, the NFκB family, and the rheumatoid arthritis and NOD-like receptor signaling pathway, compared with LPS alone (Fig. 3g and Additional file 1: Figure S6b). Together, these data suggest that ablation of NG2 glia results in a primed inflammatory response that is exaggerated following LPS challenge.

### Microglia are over-activated in the NG2 glia-ablated brain in response to LPS challenge

**Fig. 4:**
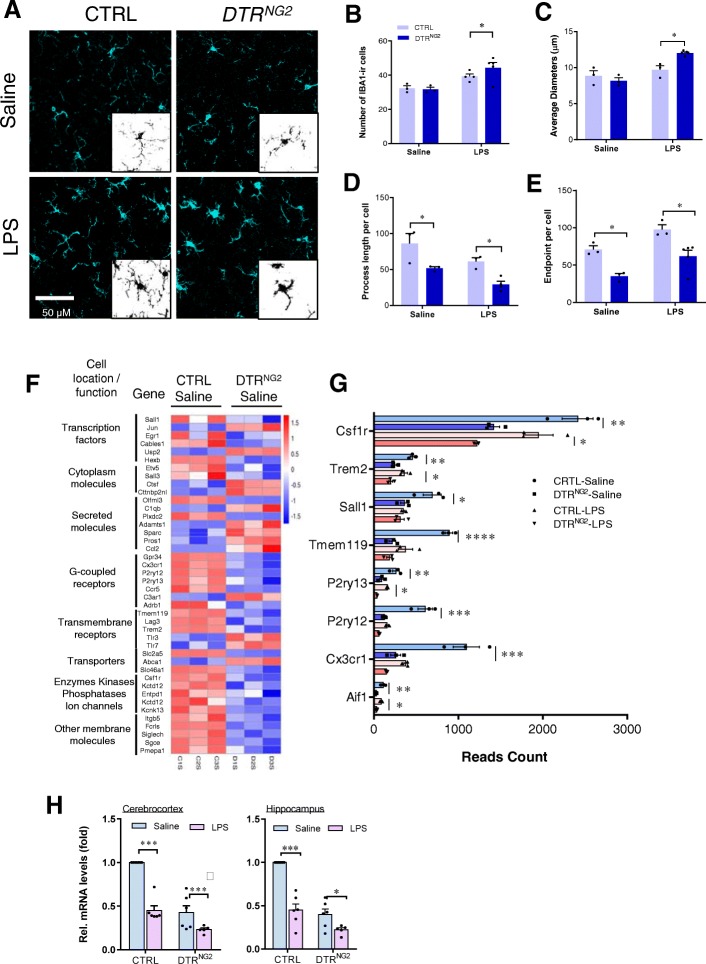
Ablation of NG2 glia results in hyper-responsiveness of microglia to LPS stimulation. **a** Immunofluorescent histochemical staining for IBA1 in the cerebrocortex of LPS-treated *DTR*^*NG2*^ Tg and control mice. Scale bars, 50 μm. **b** Quantitative estimation of IBA1-positive microglia in the data shown in **a**. **c**–**e** Quantitative assessment of IBA1-positive microglia diameters (**c**), endpoints/cell (**d**), and process length/cell (**e**) in mice with ablated NG2 glia and/or challenged with LPS. Two-way ANOVA with Tukey’s multiple comparisons test, (*n* = 3–5 mice), *P < 0.05. **f** A heatmap of microglia-enriched genes (|log2(fold change)| > 1 and *P*adj < 0.05 cutoff) regulated by ablation of NG2 glia. Profiles are shown in a color scale where blue is low and red is high. **g** Read counts of microglia-specific genes that show a twofold decrease in *DTR*^*NG2*^ saline mice compared to CTRL saline mice. *Cx3cr1*, *P2ry13*, *P2ry12*, and *Csf1r* also show a twofold decrease in *DTR*^*NG2*^ LPS mice compared to CTRL LPS mice. **h** The expression of CX3CR1 is maintained in a NG2 glia-dependent manner in adult mouse brain in vivo. qPCR analysis of the indicated brain regions, including the cerebrocortex and hippocampus of adult mice with ablated NG2 glia 4 h after systemic LPS (2.5 mg kg^−1^, i.p.) administration. Two-way ANOVA with Bonferroni’s post test (*n* = 5–6 mice per group). *P < 0.05, **P < 0.01, ***P < 0.001, ****P < 0.0001

Given that altered expression of inflammation-associated genes provoked by NG2 glia ablation is highly associated with aberrant microglial activity (Fig. 3), we addressed whether microglia in mice lacking NG2 glia displayed an activation phenotype. Quantitative morphological analysis of ionized calcium binding adaptor molecule 1 (Iba1)-positive microglia revealed that microglia in the NG2 glia-ablated brain exhibited a more intense morphological transformation after LPS challenge. Microglia in the *DTR*^*NG2*^ LPS mice became hypertrophic compared to LPS-treated WT animals (Fig. 4a–e). Likewise, the microglia *DTR*^*Olig1*^ LPS mice were also in a hyper-reactive state (Additional file 1: Figure S7) [50].

A previous study has described a set of genes that are uniquely or highly expressed in adult microglia and reduction of these genes is closed associated with microglial activation [51]. This study led us to speculate that NG2 glia may be required for the maintenance of microglia in a quiescent state via the microglial-enriched genes. Hierarchical clustering of microglial-enriched genes demonstrated that the expression of 42 microglia signature genes (log_2_ (fold change)| > 1 and *P*adj < 0.05 cutoff) were aberrantly altered in the *DTR*^*NG2*^ brain compared to the control brains (Fig. 4f). Of note, there were remarkable reductions (> 2-folds) in the expression of the microglia-specific genes, including *Aif1* (also known as *Iba1*), *Cx3cr1*, *P2ry12*, *P2ry13*, *Tmem119*, *Sall1*, *Trem2*, and *Csf1r* in the *DTR*^*NG2*^ saline mice compared to the control saline mice, as revealed by RNA-seq analysis (Fig. 4f, g). Moreover, alterations in the expression of these microglia-specific genes, including *Cx3cr1*, were validated in an independent set of samples (Fig. 4h and Additional file 1: Figure S8), indicating that NG2 glia regulates expression of microglia-specific genes and ablation of NG2 glia causes a primed and activated microglia phenotype in vivo. Furthermore, NG2 glia ablation caused profound alterations in gene expression associated with microglial M1/M2-like phenotype and it also resulted in mild upregulation of the mediators of phagocytosis (Additional file 1: Figure S9). Furthermore, the activated microglia provoked by NG2 glia ablation contributed to inflammatory response, as the levels of pro-inflammatory mediators assessed in microglia isolated from *DTR*^*NG2*^ or *DTR*^*Olig1*^ LPS mice were significantly elevated compared with LPS control animals (Additional file 1: Figure S10). These results suggest that NG2 glia are required for the maintenance of microglial homeostasis in the adult brain and CX3CR1 may play an important role in NG2 glia-regulated microglial homeostasis.

### CX3CR1 positively modulates LPS-induced neuroinflammation

**Fig. 5:**
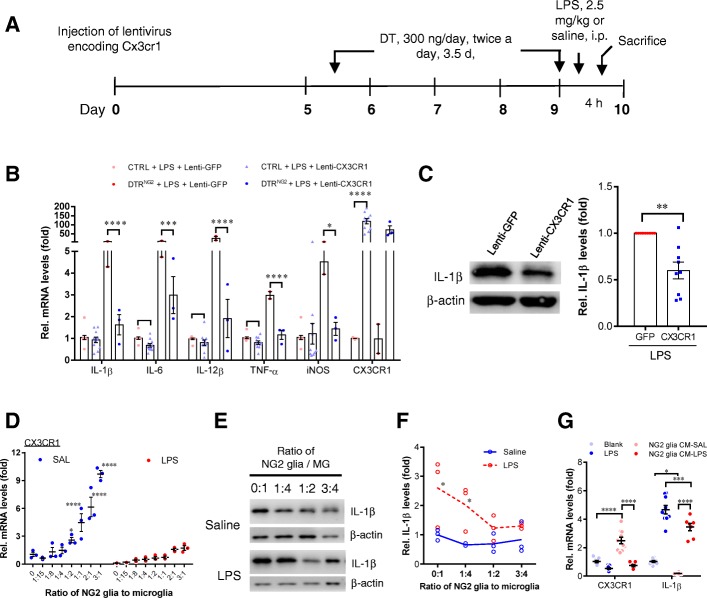
NG2 glia maintain CX3CR1 expression and attenuate LPS-induced neuroinflammation. **a** Administration paradigm used for lentivirus injection and DT/DTR-induced NG2 glia ablation in iDTR adult mouse brain. Lentivirus encoding CX3CR1 were stereotactically injected into the striatum of the *DTR*^*NG2*^ mice and counterpart control mice. The mice were maintained for 5 days after virus injection, followed by DT injection for 4 days. The mice were sacrificed after LPS (2.5 mg/kg for 4 h) challenge. **b** Representative graph showing relative mRNA levels of inflammatory mediators in the striatum from mice that received unilateral injection of lentivirus-empty vector or lentivirus-CX3CR1. Two-way ANOVA with Turkey’s post test. (*n* = 2–3 for *DTR*^*NG2*^ mice; *n* = 11 for CTRL) To analyze the effect of the CX3CR1 in the CTRL mice, two-tailed paired *t* test is used. **c** Representative western blots showing relative protein levels of IL-1ß in the striatum from mice that received unilateral injection of lentivirus-empty vector or lentivirus-CX3CR1. Paired two-tailed *t* test, (*n* = 10), **P = 0.0020. **d** Representative graph showing relative mRNA levels of CX3CR1 in co-cultures in which microglial and NG2 glial are mixed in the various ratios in the presence or absence of LPS (50 ng ml^−1^). Two-way ANOVA with Bonferroni’s post test (*n* = 5 per group). **e** Representative western blots from two separate experiments showing IL-1β expression in the co-cultures of primary NG2 glial and microglial cells at various blend ratios in the presence or absence of LPS. **f** Quantification of data shown in **e**. Two-way ANOVA with Bonferroni’s post test (*n* = 3 mice per group). **g** Transcript levels of CX3CR1 and IL-1ß evaluated by qPCR in cultured microglia which were maintained separately from NG2 glial cells in the ratio of 1:1 in the presence of NG2 glia-conditioned medium (incubated for 4 h) and LPS (50 ng ml^−1^). Data are expressed as mean ± SEM. Two-way ANOVA with Tukey’s post test (*n* = 7–10 per group). *P < 0.05, **P < 0.01, ***P < 0.001, ****P < 0.0001

Next, we investigated the role of CX3CR1 in the modulation of neuroinflammation. Previous studies have reported seemingly conflicting results regarding the effect of CX3CR1 on different brain disease pathologies [12, 52, 53]. To define the role of CX3CR1 in the context of LPS-induced neuroinflammation, we crossed *CX3CR1*^*GFP+*^ knock-in mice with each other to generate *CX3CR1* KO mice. In agreement with a previous study [12], *CX3CR1* deficiency aggravated LPS-induced neuroinflammation, as manifested by marked increases in mRNA levels of pro-inflammatory mediators (data not shown). To determine whether CX3CR1 is sufficient for the suppression of LPS-induced inflammatory response in the brain in vivo, lentivirus-mediated *CX3CR1* transfection was performed in the striatum prior to the ablation of NG2 glia and LPS exposure. Overexpression of CX3CR1 resulted in a pronounced reduction in the levels of pro-inflammatory mediators in *DTR*^*NG2*^ LPS mice compared to *DTR*^*NG2*^ LPS only mice (Fig. 5a–c), suggesting that CX3CR1 is a negative regulator of LPS-induced neuroinflammation.

We then determined whether NG2 glia are able to directly modulate microglial activity via regulating CX3CR1 expression. We employed a co-culture system in which primary NG2 glia and microglia, both derived from postnatal rat brain, were mixed at various ratios and maintained in vitro for 48 h allowing direct interactions between the two types of cells. We observed that with increasing percentages of NG2 glial cells, the levels of CX3CR1 mRNA were remarkably elevated in the co-cultures. However, the response of microglia in co-cultures was markedly blunted following exposure to LPS compared to the control (Fig. 5d). Moreover, NG2 glia are able to robustly suppress the inflammatory response of LPS-stimulated microglia. The elevation of inflammatory mediator IL-1β levels elicited by LPS was significantly antagonized by NG2 glia in a dose-dependent manner (Fig. 5e, f), as revealed by western blot analysis. Intriguingly, the observed antagonizing effect of NG2 glia was independent of cell-to-cell contact, as the effect could be at least partially mimicked by application of the conditioned medium derived from NG2 glia cultures, which also significantly enhanced CX3CR1 expression (Fig. 5g), indicating that the effects were attributed to a soluble factor(s) released by NG2 glia. These data collectively suggest that NG2 glia modulate microglial activation via upregulation of CX3CR1 expression.

### TGF-β2 derived from NG2 glia regulates CX3CR1 expression

**Fig. 6:**
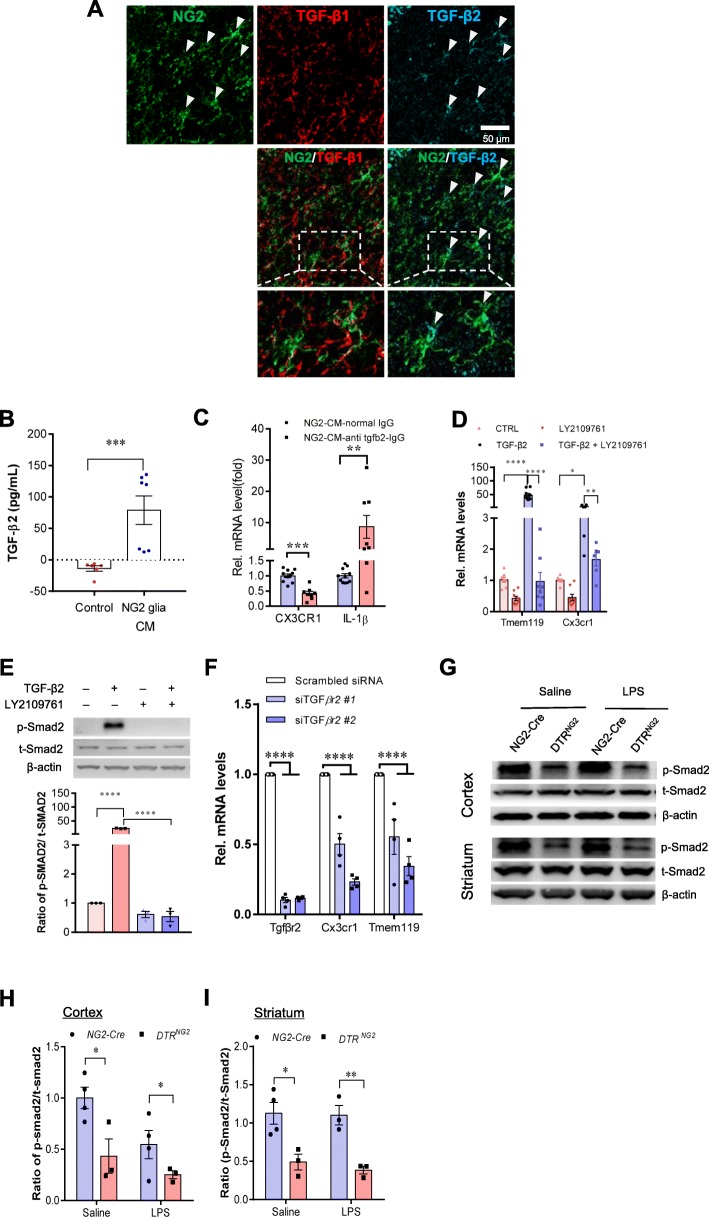
TGF-ß2/TGFBR2 signaling regulates microglial homeostasis via CX3CR1. **a** Immunofluorescent staining of representative sections showing TGF-β2 and NG2 immunoreactivities in the corpus callosum of wild-type mice. Arrows indicate double-labeled cells. Scale bar, 50 μm. **b** Measurement of TGF-β2 protein levels was performed in the concentrated conditioned medium (CM) from primary NG2 glial cells using TGF-β2 ELISA (*n* = 6 for control medium, *n* = 7 for NG2 glia CM). Unpaired *t* test. **P < 0.01. **c** Pre-incubation of NG2 CM with antibody against TGF-ß2 markedly reduces the mRNA levels of CX3CR1 and increases IL-1β expression in primary cultured microglia. Unpaired two-tailed *t* test, (*n* = 8–10). ***P < 0.001 for CX3CR1. Nonparametric test with Mann–Whitney test, **P < 0.01 for IL-1β. All data are expressed as mean ± SEM. **d** qPCR analysis shows that the increases in the expression of Tmem119 and CX3CR1 in microglial cell line BV2 cells were abolished upon addition of LY2109761, a TGFBR1/2 blocker. One-way ANOVA with Newman–Keul’s post test (6–8 independent experiments). *P < 0.05, **P < 0.01, ****P < 0.001. **e** Immunoblotting analysis reveals that the elevations in the levels of phopho-Smad2 evoked by TGF-β2 are blocked by LY2109761 in BV2 cells. One-way ANOVA with Newman–Keul’s post test (*n* = 3 independent experiments). ****P < 0.001. **f** qPCR analysis shows that the decreases in the expression of CX3CR1 and Tmem119 in BV2 cells were induced upon transfection of TGFBR2 siRNA #1 or #2. One-way ANOVA with Newman–Keul’s post test (*n* = 4 independent experiments). ****P < 0.001. **g** Representative western blots from two separate experiments showing the decreases in the levels of phopho-Smad2 in both the cerebrocortex and striatum of mice with ablated NG2 glia. **h**, **i** Quantification of data shown in **g**. Two-way ANOVA with Bonferroni’s test (*n* = 3 mice per group). *P < 0.05, **P < 0.01

We then determined the molecular basis underlying the NG2 glial control of microglial activation. To clarify the molecular identity of the soluble factor(s), we examined the association between NG2 glia and TGF-β family members, given that TGF-β signaling has been shown to suppress the activation and proliferation of microglia in vitro [51, 54, 55], although the exact molecular mechanisms underlying the inhibitory effects by TGF-β on microglia have not been fully elucidated. Immunohistochemical double labeling staining showed that the immunosignals for TGF-β2, but not TGF-β1, were co-localized in some NG2^+^ cells in the adult brain (Fig. 6a). The enzyme-linked immunosorbent assay (ELISA) data showed that the immunosignals for TGF-β2 (Fig. 6b) were detectable in the conditioned medium of NG2^+^ cells compared with control, indicating that TGF-β2 is enriched in NG2 glia.

We next sought to assess the impact of TGF-β2 on the maintenance of microglia quiescence. As shown in Fig. 6c, neutralization of TGF-β2 activity in NG2 glia-derived conditioned medium with a specific monoclonal TGF-β2 antibody significantly downregulated CX3CR1 expression and consequently promoted IL-1β expression in primary cultured microglia, supporting the notion that NG2 glia modulate microglial activity via TGF-β2. We found that the modulatory effect of TGF-β2 was dependent on the microglial TGF-β type II receptor (TGFBR2). Treatment of primary microglial cell cultures with TGF-β2 resulted in remarkable increases in mRNA levels of CX3CR1 and Tmem119, two microglial checkpoint genes, compared to the control. The effects, however, were abolished by co-treatment with LY2109761, a selective TGFBR1/2 inhibitor (Fig. 6d), and were accompanied by dramatic downregulation of the phosphorylation of Smad2, a key component of the TGF-β receptor signaling cascade (Fig. 6e), indicating that microglial TGF-β receptor signaling plays an important role in the process of maintaining microglia quiescence. Moreover, knockdown of TGFBR2 in primary microglia confirmed the observation that TGFBR2 deficiency in microglia drastically suppressed the mRNA expression of CX3CR1 and Tmem119 (Fig. 6f). Furthermore, dysfunction of TGF-β receptor/SMAD2 signaling was also observed in the cerebrocortex and striatum of the brain with ablation of NG2 glia in vivo (Fig. 6g–i). These data suggest that TGF-β receptor/SMAD2 signaling is involved in the regulation of microglia homeostasis.

In alignment with this, we found that augmentation of TGF-β signaling with TGF-β2 in a murine microglial cell line BV2 caused a drastic increase in the levels of multiple microglial checkpoint gene transcripts, including *P2ry12*, *P2ry13*, *Csf1r*, *Sall1*, and *TGFBR1*. Conversely, blockade of TGFBR2 signaling with either LY2109761 treatment or siRNA-mediated TGFBR2 knockdown resulted in a remarkable decrease in expression of the microglial checkpoint gene (Additional file 1: Figure S11). Taken together, NG2 glia-activated TGFβ2-TGFBR2 signaling is crucial for the maintenance of CX3CR1 expression thereby controlling microglial homeostasis.

### Activation of TGF-β2/TGFBR2 signaling inhibits LPS-induced neuroinflammation

**Fig. 7:**
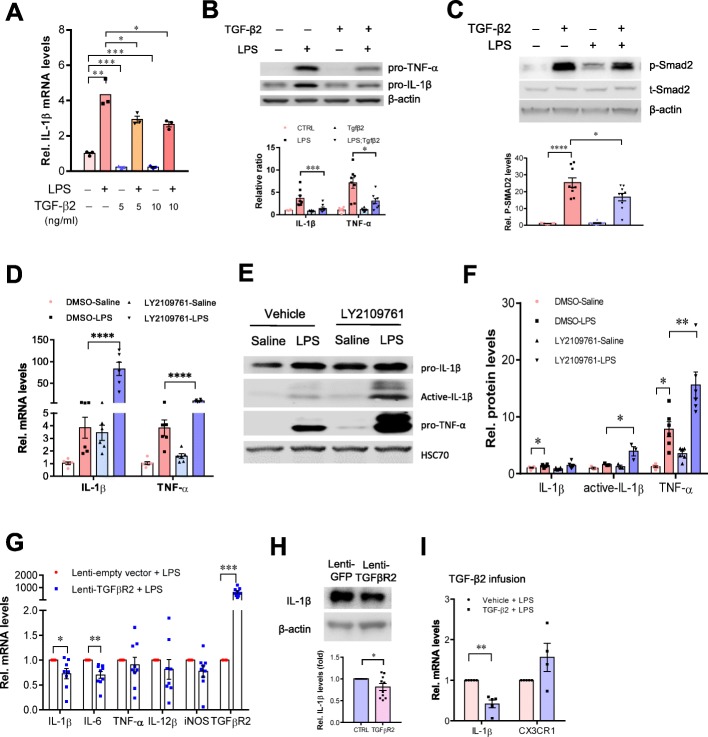
Activation of TGF-β2/TGFBR2 signaling attenuates LPS-induced neuroinflammation. **a** qPCR analysis showing the reduction of IL-1β mRNA levels in primary cultured microglia treated with TGF-β2 (5, 10 ng ml^−1^, 4 h) in the presence or absence of LPS (50 ng ml^−1^). Unpaired *t* test (*n* = 3 independent experiments). **b**, **c** Representative immunoblots showing the increases in the levels of IL-1β and TNF-a protein induced by LPS were attenuated by TGF-β2 treatment in BV2 cells (*n* = 8 independent experiments). This process is accompanied by alterations in the levels of phospho-SMAD2 (**c**). *n* = 9 independent experiments. **d** qPCR analysis shows that the disruption of TGFBR2 signaling by LY2109761 exacerbates inflammatory response to LPS stimulation. BV2 cells were treated with LY2109761 (10 ng/ml) or vehicle DMSO for 2 h, followed by exposure to LPS (100 ng/ml) or saline for 6 h. Two-way ANOVA with Turkey’s test (*n* = 6 independent experiments). **e**, **f** Immunoblot analysis showing the increases in the levels of active-IL-1β in BV2 cells with disruption of TGFBR2 signaling by LY2109761. **f** Quantification of data shown in **e**. Two-way ANOVA with Turkey’s test. Data are expressed as mean ± SEM (*n* = 3–6). *P < 0.05, **P < 0.01, ***P < 0.001. **g** qPCR analysis of the striatum of wild-type mice that received stereotactic injection of lentivirus-TGFBR2 showed that the production of inflammatory mediators was attenuated by TGFβR2 overexpression. Unpaired *t* test (*n* = 8–9). *P < 0.05, **P < 0.01, ***P < 0.001. ***P < 0.001. **h** Representative western blots showing the decreases in levels of IL-1β in the striatum of mice that received stereotactic injection of lentivirus-TGFBR2. The striatum in contralateral side received injection of lentivirus-GFP served as control. Data are expressed as mean ± SEM (*n* = 10). Paired *t* test. *P < 0.05. **i** qPCR analysis shows the alterations in mRNA levels of IL-1β and CX3CR1 in the brain that received intracerebroventricular infusion of active form of TGF-β2. The animals received single injection of LPS (2.5 mg/kg, i.p.) 1 h after TGF-β2 infusion. Unpaired *t* test (*n* = 4–5 mice per group). **P < 0.01

Next, we evaluated the impact of TGF-β2/TGFBR2 signaling on the modulation of neuroinflammation. Exposure of the primary cultured microglia to TGF-β2 decreased the levels of pro-inflammatory mediators provoked by LPS treatment (Fig. 7a). Consistent with these results, TGF-β2 exhibited a potent anti-inflammatory effect in LPS-treated BV2 cells, which was accompanied by marked alterations in the levels of Smad2 phosphorylation (Fig. 7b, c). Blockade of TGF-β2/TGFBR2 signaling in cultured BV2 cells by treatment with LY2109761 resulted in further increases in the levels of pro-inflammatory mediators compared with LPS treatment alone, as revealed by qPCR and western blotting analysis (Fig. 7d–f).

Moreover, augmentation of TGFBR2 signaling by lentivirus-mediated TGFBR2 gene transfection into the striatum led to a decrease in the levels of pro-inflammatory mediators elicited by LPS (Fig. 7g, h), indicating that TGF-β2/TGFBR2 signaling is important for control of microglia-mediated neuroinflammation in the CNS in vivo. This finding was further supported by the observation that TGF-β2 protein infusions into the dorsal striatum caused a profound decrease in the levels of IL-1β mRNA, which was accompanied by a trend toward an increase in the levels of CX3CR1 mRNA in the striatum of wild-type mice in vivo compared with LPS-treated animals (Fig. 7i). Altogether, these data suggest that NG2 glia inhibit aberrant activation of microglia via TGF-β2/TGFBR2/CX3CR1 signaling.

### Dysfunction of NG2 glia exacerbates microglial activation and nigral dopaminergic neuron loss

**Fig. 8:**
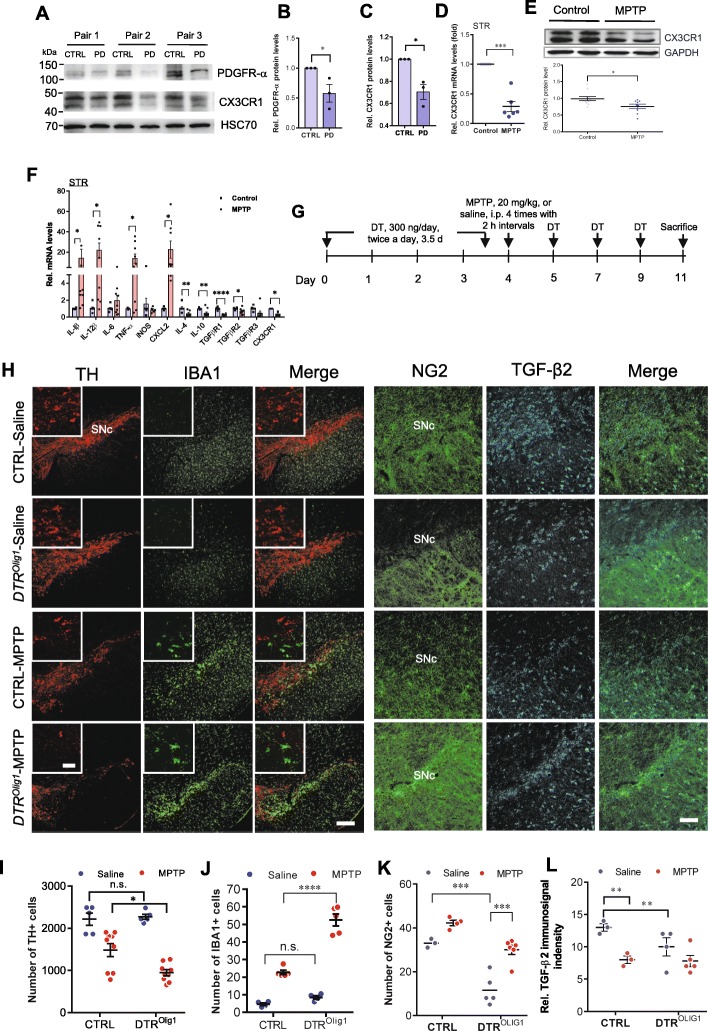
NG2 glia deficiency contributes to neurodegeneration in both patients with Parkinson’s disease and MPTP-induced mouse PD model. **a**–**c** Downregulation of PDGFRα and CX3CR1 expression in the substantia nigra of patients with PD compared with control subjects. Student’s *t* test (*n* = 3). **d**–**f** Ablation of NG2 glia exacerbates microglial activation and nigral dopaminergic neuron loss elicited by MPTP treatment. **d**, **e** Downregulation of microglia CX3CR1 in the striatum of MPTP-induced PD model as revealed by qPCR analysis (**d**) or immunoblotting analysis (**e**). The mice were sacrificed 24 h following MPTP exposure (*n* = 3 per group). **f** Representative graph showing the mRNA levels of pro-inflammatory mediators and TGFBR1-3 in the CD11b-positive microglia isolated from the striatum of MPTP-induced PD model using MACS. Student’s *t* test, *paired*, (*n* = 6). *P < 0.05, ***P < 0.001*.* **g**–**l** Ablation of NG2 glia exacerbates microglial activation and nigral dopaminergic neuron loss elicited by MPTP treatment. **g** Schematic representation of the generation of *DTR*^*NG2*^ Tg mice and MPTP-induced PD mouse model. DT (300 ng/day) was given twice a day for 3.5 days. MPTP was given at 20 mg/kg for four times with 2-h intervals following NG2 glia ablation. **h** Immunofluorescent histochemical analysis of the ventral midbrain sections taken 7 days from *DTR*^*Olig1*^ mice and littermate control that were challenged with MPTP. **i**–**l** Quantitative data shown in **h**. Two-way ANOVA with Bonferroni’s post test; (*n* = 3–6). *P < 0.05, **P < 0.01; ****P < 0.0001, ****P < 0.0001. Scale bars, 200 μm; scale bar in the inserts, 50 μm

We then asked whether dysregulation of NG2 glia-controlled immune homeostatic process contributes to neurodegeneration of nigral dopaminergic (DA) neurons which is associated with prominent neuroinflammation [5]. To investigate the pathological relevance of these findings, we examined the expression of PDGFRα, markers of NG2 glia [15], and CX3CR1 in the brain of patients with PD. We found a profound downregulation of their expression in the SN of PD brain compared with healthy subjects (Fig. 8a–c), indicating dysfunction of NG2 glia and compromised microglial homeostatic state in PD brain. Moreover, in an acute MPTP mouse model which is a well-accepted model of the disease, there was a marked decrease in the levels of TGFBR1, TGFBR2, CX3CR1, and anti-inflammatory mediators, including IL-4 and IL-10 in the striatum (Fig. 8d–f). The imbalance of brain innate immunity provoked by MPTP treatment was accompanied by a pronounced increase in the levels of pro-inflammatory mediators, including IL-1β, TNF-α, and CCL-2 (Fig. 8f).

Microglial neurotoxicity has been proposed to augment the severity of neurodegenerative processes of nigral DA neurons. To this end, we investigated a potential role for NG2 glia in the microglial activation to neurotoxin MPTP, which selectively induces degeneration of DA neurons, by ablating NG2 glia in *DTR*^*Olig1*^ mice (Fig. 8g). Cell counting of the SNc of saline- and MPTP-treated *DTR*^*Olig1*^ and control (*Olig1-Cre* only) littermate mice 7 days after challenge showed that the loss of tyrosine hydroxylase–immunoreactive (TH+) cells in the SN was significantly worse (~ 58%) in NG2 glia-depleted mice following MPTP exposure compared to MPTP-treated control animals (~ 33% reduction) (Fig. 8h, i). Further examination of coronal sections of the SN of *DTR*^*Olig1*^ mice revealed a significant increase (~ 2.3-fold) in the number of microglia and more prominent and restricted NG2^+^ cell activation (glial scar) in the SNc, as compared to those in control animals, in response to systemic administration of MPTP (Fig. 8h–k). Interestingly, TGF-β2 immunoreactivity was significantly reduced in NG2 glia-ablated mice with or without MPTP administration (Fig. 8h, l and Additional file 1: Figure S12). Together, these data suggest that NG2 glia are required for the suppression of microglial activation in PD pathogenesis.

## Discussion

In the present study, we showed the fundamental role of quiescent NG2 glia in the cellular network that controls the innate immunity balance in the adult mouse brain and their contribution to PD pathogenesis. These NG2 glia are both necessary and sufficient for microglial maintenance in a quiescent state under physiological conditions. NG2 glia also contribute to the suppression of over-activation of microglia evoked by immune stimuli and MPTP-induced neurotoxicity. TGF-β2 profoundly influences the expression of CX3CR1 thereby determining the response of microglia to immune stimuli. In line with this, TGF-β2 antagonizes the negative effect of LPS on the expression of CX3CR1 in microglia. Moreover, NG2 cells are neuroprotective to DA neurons in MPTP-induced PD model possibly via TGF-β2. Thus, our data have demonstrated a novel molecular and cellular mechanism for the regulation of innate immunity in the adult brain and the significant contribution in MPTP-induced PD model (Additional file 1: Figure S13).

One of the most interesting findings in the present study is that the lack of quiescent NG2 glia in adult animals perturbs the microglial homeostasis, highlighting the crucial role of NG2 glia–microglial communication in maintaining the resting state of microglia. One of the intriguing questions in the field of glial biology has been what the physiological functions of NG2 glia are in the adult brain, where the process of myelination is largely complete and the NG2 glia become much less active than in postnatal life [56–58]. The present findings suggest that, in addition to being oligodendrocyte precursor cells involved in remodeling existing myelin [58] or remyelination after brain injury, NG2 glia have a novel function in controlling immune homeostasis which is essential for maintaining normal functionality of the adult CNS. Indeed, in the present study, NG2 glial regulation of microglial function was observed in the four major brain regions examined, including the cerebrocortex, hippocampus, striatum, and SNc, indicating that NG2 glia exert a function as an immunosuppressor throughout the entire mouse brain. This assumption is supported by the evidence that NG2 glia exist broadly in the white and gray matter of the adult CNS and are almost as numerous as astrocytes [59], suggesting that the newly revealed function for NG2 glia in this study is highly correlated with its distribution pattern in the CNS. This observation indicates that NG2 glial control of microglial activity may represent a universal mechanism of maintaining the delicate immune balance across the entire CNS.

It is well established that, in the adult brain, microglia are capable of rapidly “reacting” to injuries and have a strong potential to proliferate and migrate to areas of lesions [12, 60, 61]. In the present study, we found that microglia were transformed into a mild reactive state in the absence of NG2 glia, since among the pro-inflammatory mediators examined, the levels of TNF-α were moderately increased in the brain with ablated NG2 glia, which was accompanied by marked downregulation of multiple microglia checkpoint genes under basal conditions. Moreover, the combination of NG2 glia ablation and LPS challenge significantly activated pro-inflammatory signaling pathways, such as cytokine and cytokine receptor interactions, the toll-like receptor signaling pathway, the Tnf signaling pathway, the NFκB family, and the rheumatoid arthritis and NOD-like receptor signaling pathway (Fig. 3g), indicating that NG2 glia play a vital role in the suppression of inflammatory response. These findings support the notion that there is a close spatial relationship between the NG2 glia and microglia under the normal and pathological conditions [62]. For instance, in systemic inflammation, microglia may exert a negative effect on NG2 glia activity [63] promoting an inflammatory response. Moreover, the perturbed microglial homeostasis appears to result exclusively from the NG2 glia depletion, as selective ablation of mature oligodendrocytes, which are derived from NG2 glia during brain development, failed to significantly alter the immune balance in the brain (Fig. 2d). These data suggest that acute ablation of NG2 glia, but not diphtheria toxin-induced cell death itself, primes the microglia for inflammation. Thus, it is conceivable that NG2 glia, but not mature oligodendrocytes belonging to the same cell lineage, specifically regulate brain innate immunity via controlling the microglial silenced state.

Emerging evidence indicates that the maintenance of microglia in a quiescent state is regulated through a set of microglial-enriched genes. Much has been learned in recent years about the identity of these genes. Thus, several of these genes, such as *Olfm13* [51], *Tmem119* [51], *Sall1* [51, 64], and *Cx3cr1* [65], have been identified. Indeed, in the present study, we observed significant downregulation of these genes in the NG2 glia-ablated brain. However, the biological function and significance of these genes in the maintenance of microglial homeostasis remains largely unknown. Moreover, the upstream factors critical for the regulation of microglial quiescence under physiological conditions have not been fully elucidated. A recent study showed that TGF-β signaling is required for the in vitro development of microglia. Specifically, TGF-β1 is necessary for microglia survival, as microglia were absent in the CNS of TGF-β1-deficient mice [51]. In contrast, we showed in the present study that TGF-β2 is a major factor released from NG2 glia and plays an important role in the microglial maintenance via CX3CR1, indicating that NG2 glia are critical components regulating microglial homeostasis under physiological conditions.

NG2 glia–microglial communication represents a unique cellular mechanisms as a part of the neural networks that tightly controls microglia activity, in addition to the neuron–microglial and astrocyte–microglial interactions that have been relatively well documented [66, 67]. It is worth noting that NG2 glia may modulate neuroinflammation in a NG2 gene-independent manner. Previous studies by others showed that traumatic brain injury-induced expression of pro-inflammatory cytokine genes was unchanged between NG2 gene knockout and control animals [68]. This observation is different from the findings in the present study in which the NG2 glia-ablated mouse brain displayed remarkable increases in pro-inflammatory mediators following exposure to pathological stimuli.

With the identification of additional microglia-enriched genes, a molecular microglial signature is now often used to describe the features that account for microglia homeostatic status. We need to better characterize the beneficial versus detrimental properties of these genes under pathological conditions [51, 69]. Ample concrete evidence has shown that CX3CR1 is involved in inflammatory processes and is required for the balance of microglial activation [12, 70]. Whether CX3CR1 is sufficient for the suppression of neuroinflammation remains elusive. We demonstrated that overexpression of CX3CR1 was able to attenuate the inflammatory response provoked by LPS in the NG2 glia-ablated brain compared to LPS treatment alone, which supports a previous study that at least in context of LPS-induced neuroinflammation, CX3CR1 is one of key players in suppression of microglia-driven neuroinflammation. These data indicate that control of the activation status of microglia via CX3CR1 is crucial for suppression of neuroinflammation.

How is CX3CR1 expression regulated? Accumulating evidence suggests that regulation of CX3CR1 expression is considerably complex in vivo, as CX3CL1 signaling may be either positively or negatively regulated in distinct contexts, such as LPS challenge, experimental autoimmune encephalomyelitis (EAE), multiple sclerosis, and HIV infection [71]. In addition to microglia, much evidence supports the expression of CX3CR1 in non-microglial cells, such as oligodendrocyte precursor cells (OPCs), neural precursors and neurons, although to a lower extent [72–76]. We showed in Fig. 7i that TGF-β2 did not significantly elevate CX3CR1 expression levels in LPS conditions. However, LPS injection led to an increase in Olig1 expression in Olig1-Cre animals, as shown in Additional file 1: Figure S4. Thus, it is possible that LPS injection in WT animals can lead to an increase in CX3CR1 levels in oligodendroglial lineage cells, which can in turn contribute to variability in non-microglial CX3CR1 expression in LPS and TGF-β2 injected animals, given that other cell types also express CX3CR1, especially because OPCs can themselves express CX3CR1 [72].

The TGF-β family consists of three closely related members, TGF-β1, TGF-β2, and TGF-β3, which exert diverse functions, including regulation of adaptive immunity, inhibition and stimulation of cell proliferation, and neuroprotection [77]. TGF-β triggers signaling via binding to the TGF-β receptor complex which is a tetrameric structure, composed of two type I TGF-β receptors (TGFBR1) and two TGFBR2. Both TGFBR1 and TGFBR2 are highly expressed on microglia compared with spleen macrophage [78]. Our study revealed that the TGF-β2/TGFBR2 signaling pathway significantly contributes to the inhibitory effects of NG2 glia on microglial activation in the adult mouse brain via the increase in SMAD2 phosphorylation leading to the elevation of CX3CR1 expression. These observations are consistent with recent findings that dysfunction of TGFβ signaling in microglia results in impaired homeostasis [50, 79]. Thus, TGF-β2 is a critical factor mediating NG2 glia–microglial interactions thereby keeping microglia in a steady state.

The present study showed that ablation of NG2 glia led to more severe loss of DA neurons and enhanced microglial activation caused by MPTP neurotoxicity, indicating that NG2 glia plays an important role in the modulation of inflammatory processes of PD pathology. Thus, our findings highlight the therapeutic potential of NG2 glia in inflammation-associated brain disorders. Recent studies have shown that the proliferative ability of NG2 glia diminishes with aging [31, 80], and NG2 glia are correlated with the pathogenesis of Alzheimer’s disease [24]. It is likely that dysfunction of NG2 glia compromises NG2 glia–microglia communication and undermines the capacity for suppression of neuroinflammation. Consequently, microglia enter a partially reactive state. This progression may help explain why the aging brain becomes susceptible to neurodegenerative diseases.

## Conclusions

In summary, this study reveals a novel function for NG2 glia as suppressors for neuroinflammation in the CNS. Strategies to maintain the number or function of NG2 glia in the CNS directly or by strengthening NG2 glia–microglia communication may thus have therapeutic utility in inflammation-associated brain disorders. Thus, the findings in the present study open up new paths in the study of broader range of brain disorders associated with neuroinflammation.

## Supplementary information

**Additional file 1:** Supplementary figures.

## Data Availability

The data supporting the conclusions of this article are available from the corresponding author upon request.

## Abbreviations

CX3CR1: CX3C chemokine receptor 1
DA: Dopaminergic
DTR: Diphtheria toxin receptor
ELISA: Enzyme-linked immunosorbent assay
GFAP: Glial fibrillary acidic protein
IL: Interleukin
LPS: Lipopolysaccharide
MPTP: 1-Methyl-4-phenyl-1,2,3,6-tetrahydropyridine
NG2: Neuron-glial antigen 2
OPC: Oligodendrocyte precursor cell
PD: Parkinson’s disease
PLP1: Proteolipid protein 1
TGF-β: Transforming growth factor-β
TGFBR2: TGF-β type II receptor
