# Supplementary material for: NG2 glia regulate brain innate immunity via TGF-β2/TGFBR2 axis
Source: BMC Med. 2019 Nov 15;17:204. doi: 10.1186/s12916-019-1439-x (PMC6857135; doi:10.1186/s12916-019-1439-x)
Supplement: Supplementary file 1 — Additional file 1: Supplementary figures. [file 12916_2019_1439_MOESM1_ESM.zip › 191109 Figures_Suppl_ updated.pdf]

Figure S1

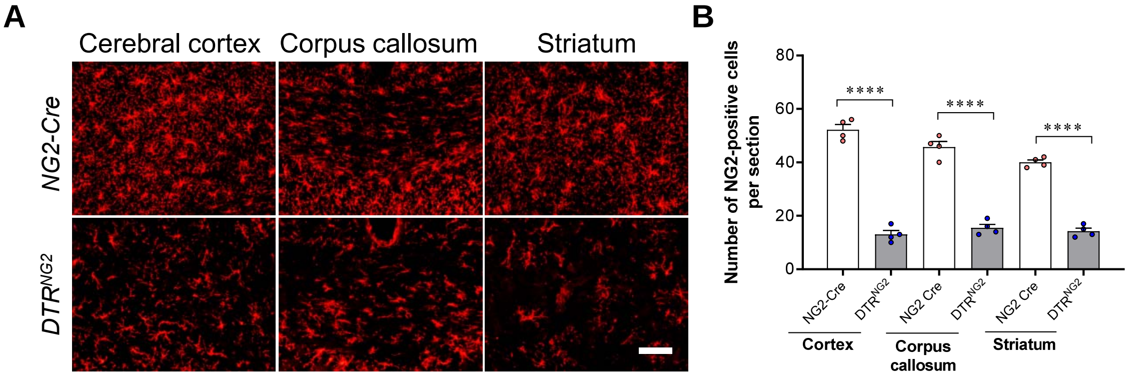

A
Cerebral cortex
Corpus callosum
Striatum
NG2-Cre
DTR^NG2
B
Number of NG2-positive cells per section
80
60
40
20
0
****
****
****
NG2-Cre
DTR^NG2
NG2 Cre
DTR^NG2
NG2 Cre
DTR^NG2
Cortex
Corpus callosum
Striatum

Figure S2

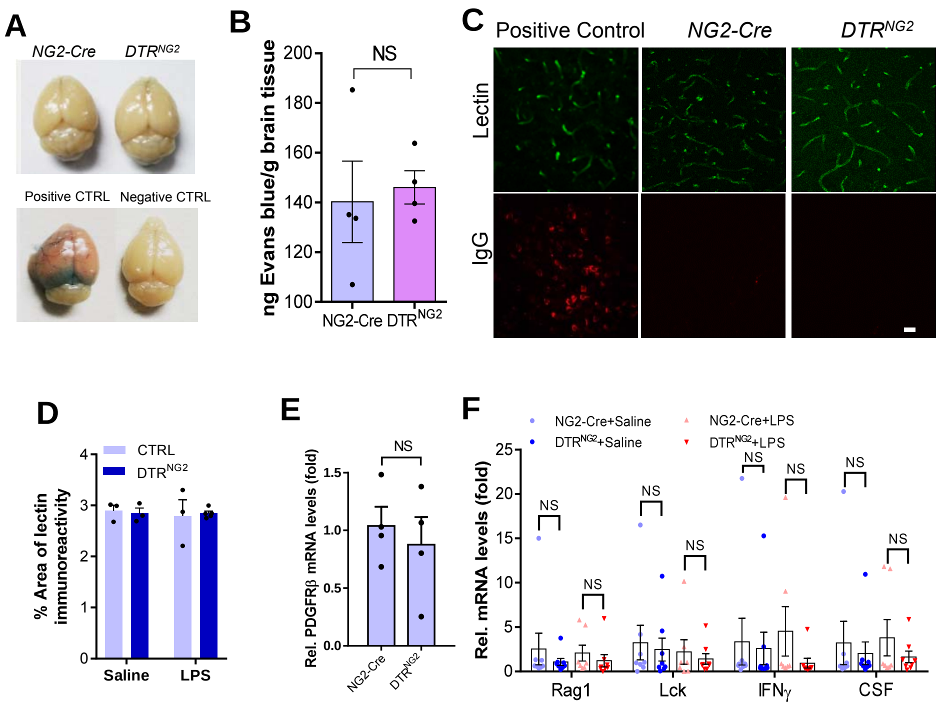
A
NG2-Cre
DTR^NG2
Positive CTRL
Negative CTRL
B
NS
ng Evans blue/g brain tissue
200
180
160
140
120
100
NG2-Cre
DTR^NG2
C
Positive Control
NG2-Cre
DTR^NG2
Lectin
IgG
D
CTRL
DTR^NG2
% Area of lectin immunoreactivity
4
3
2
1
0
Saline
LPS
E
NS
Rel. PDGFRβ mRNA levels (fold)
1.5
1.0
0.5
0.0
NG2-Cre
DTR^NG2
F
NG2-Cre+Saline
DTR^NG2+Saline
NG2-Cre+LPS
DTR^NG2+LPS
Rel. mRNA levels (fold)
25
20
15
10
5
0
NS
Rag1
Lck
IFNγ
CSF

Figure S3

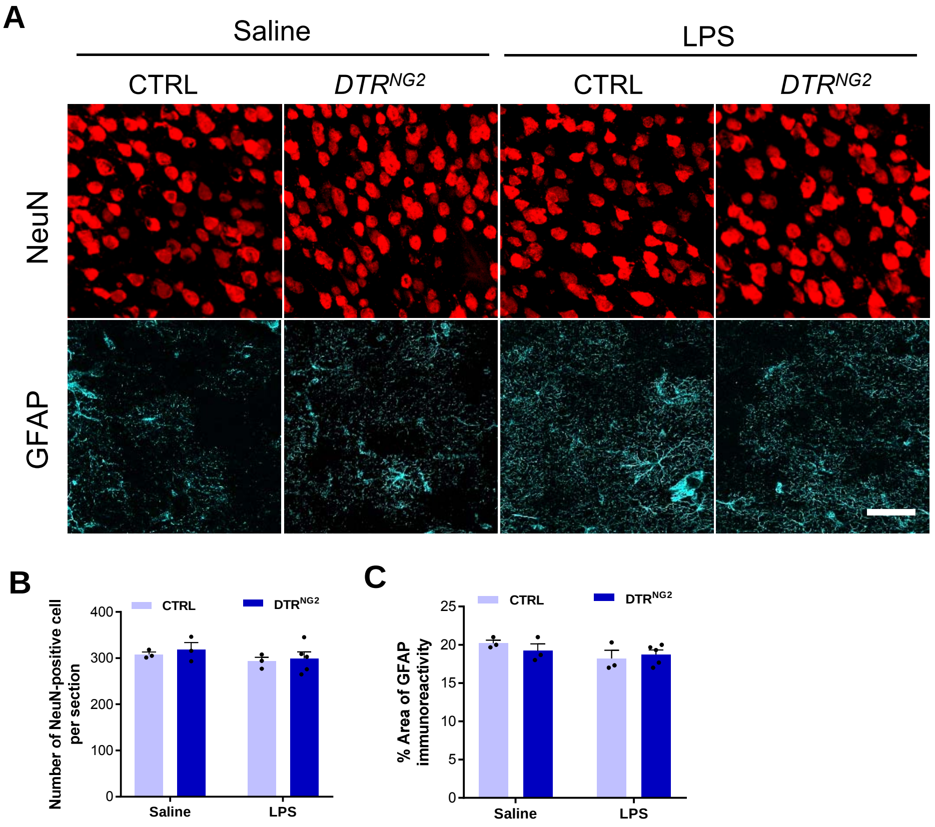
A
Saline
LPS
CTRL
DTR^NG2
CTRL
DTR^NG2
NeuN
GFAP
B
CTRL
DTR^NG2
Number of NeuN-positive cell per section
400
300
200
100
0
Saline
LPS
C
CTRL
DTR^NG2
% Area of GFAP immunoreactivity
25
20
15
10
5
0
Saline
LPS

Figure S4

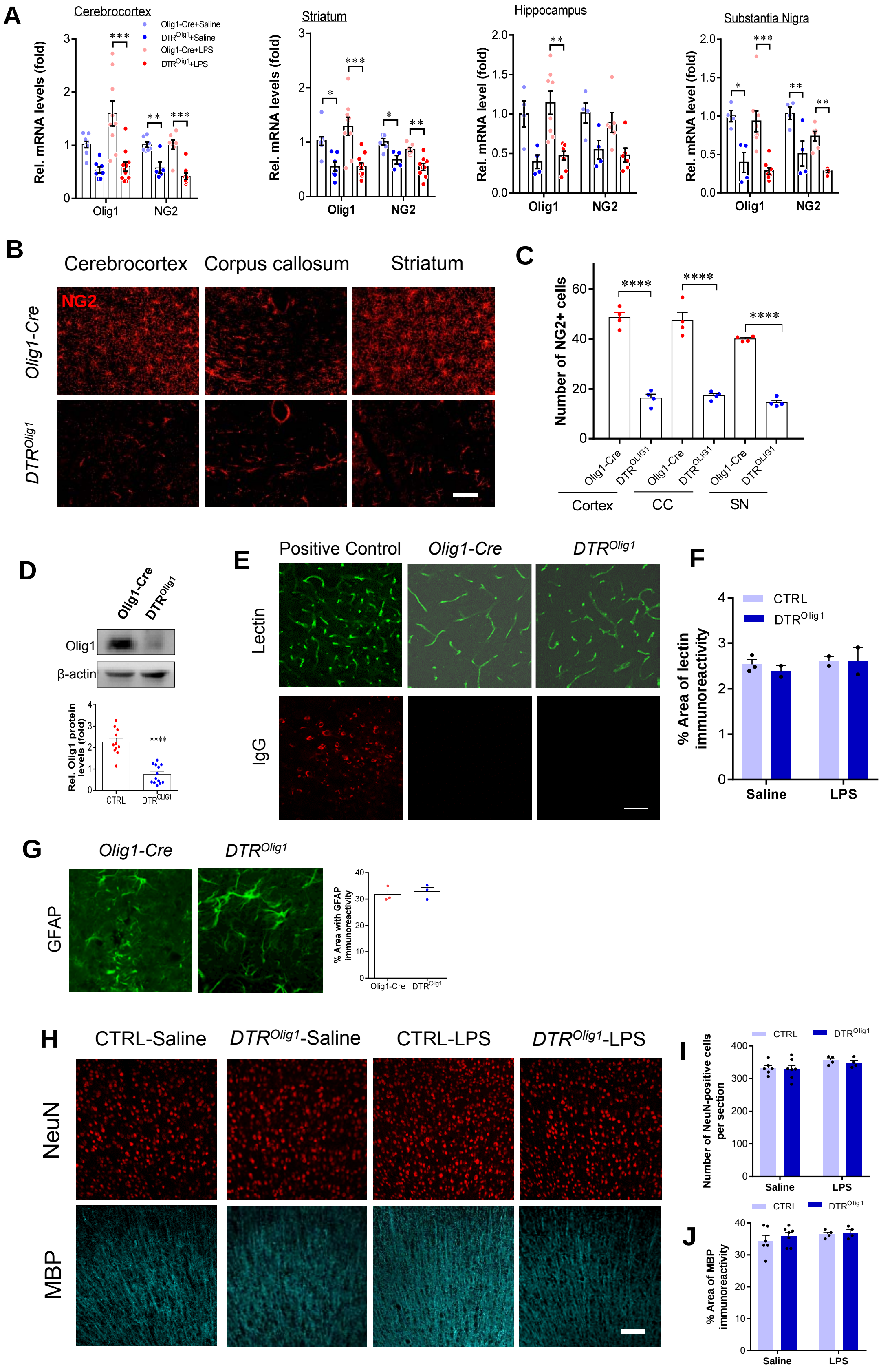
A
Cerebrocortex
Olig1-Cre+Saline
DTR^Olig1+Saline
Olig1-Cre+LPS
DTR^Olig1+LPS
Rel. mRNA levels (fold)
Olig1
NG2
Striatum
Hippocampus
Rel. mRNA level (fold)
Substantia Nigra
B
Cerebrocortex
Corpus callosum
Striatum
NG2
Olig1-Cre
DTR^Olig1
C
Number of NG2+ cells
Olig1-Cre
DTR^OLIG1
Cortex
CC
SN
D
Olig1-Cre
DTR^Olig1
Olig1
β-actin
Rel. Olig1 protein levels (fold)
CTRL
DTR^OLIG1
E
Positive Control
Olig1-Cre
DTR^Olig1
Lectin
IgG
F
CTRL
DTR^Olig1
% Area of lectin immunoreactivity
Saline
LPS
G
Olig1-Cre
DTR^Olig1
GFAP
% Area with GFAP immunoreactivity
H
CTRL-Saline
DTR^Olig1-Saline
CTRL-LPS
DTR^Olig1-LPS
NeuN
MBP
I
Number of NeuN-positive cells per section
J
% Area of MBP immunoreactivity

Figure S5

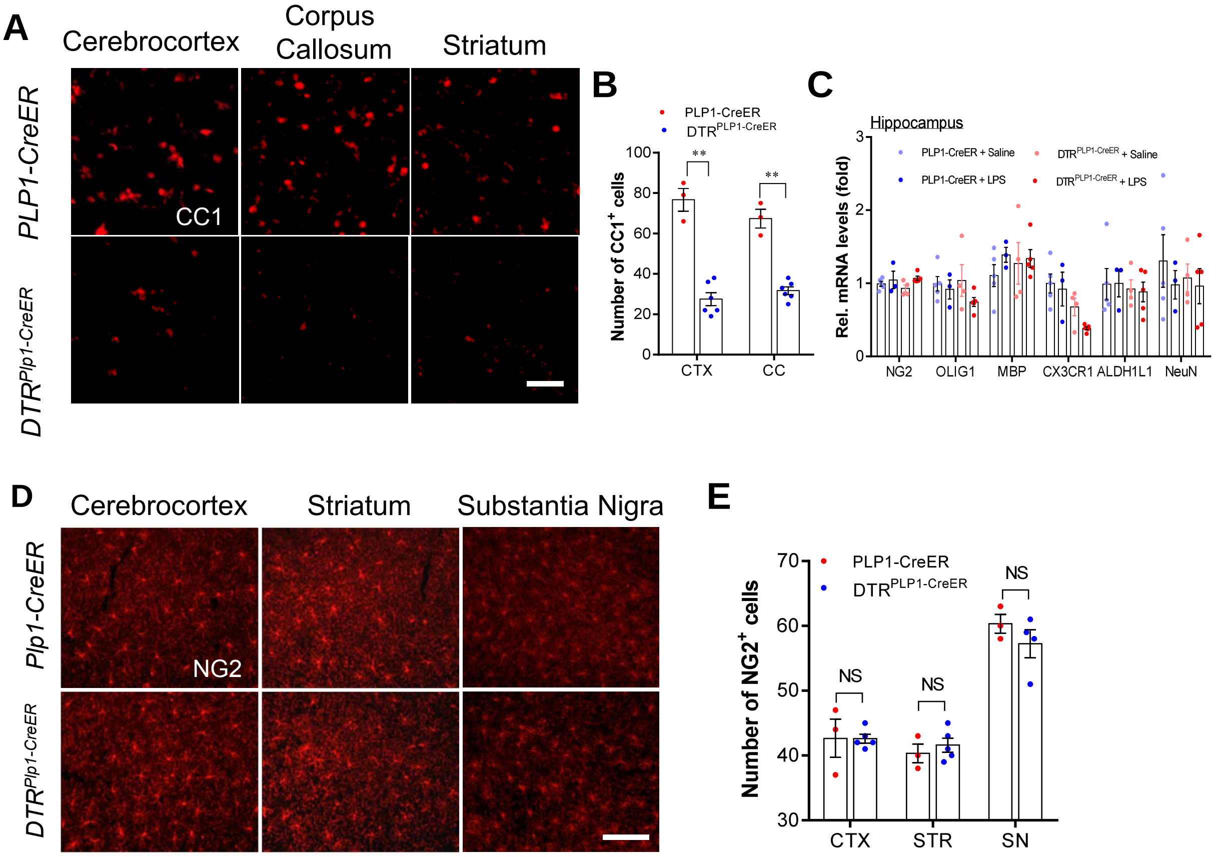

A
Cerebrocortex
Corpus Callosum
Striatum
PLP1-CreER
DTR^Plp1-CreER
CC1
B
PLP1-CreER
DTR^PLP1-CreER
Number of CC1+ cells
**
CTX
CC
C
Hippocampus
PLP1-CreER + Saline
DTR^PLP1-CreER + Saline
PLP1-CreER + LPS
DTR^PLP1-CreER + LPS
Rel. mRNA levels (fold)
NG2
OLIG1
MBP
CX3CR1
ALDH1L1
NeuN
D
Cerebrocortex
Striatum
Substantia Nigra
Plp1-CreER
DTR^Plp1-CreER
NG2
E
PLP1-CreER
DTR^PLP1-CreER
Number of NG2+ cells
NS
CTX
STR
SN

Figure S6

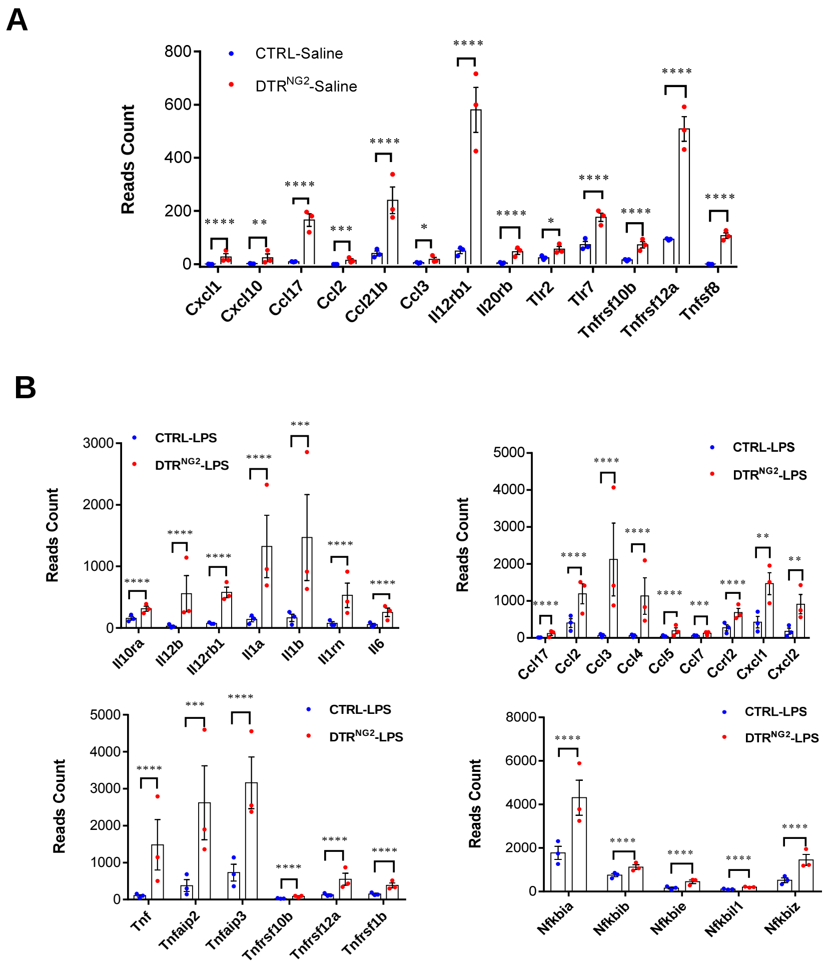

A
CTRL-Saline
DTRNG2-Saline
Reads Count
Cxcl1
Cxcl10
Ccl17
Ccl2
Ccl21b
Ccl3
Il12rb1
Il20rb
Tlr2
Tlr7
Tnfrsf10b
Tnfrsf12a
Tnfsf8
B
CTRL-LPS
DTRNG2-LPS
Reads Count
Il10ra
Il12b
Il12rb1
Il1a
Il1b
Il1rn
Il6
Ccl17
Ccl2
Ccl3
Ccl4
Ccl5
Ccl7
Ccrl2
Cxcl1
Cxcl2
Tnf
Tnfaip2
Tnfaip3
Tnfrsf10b
Tnfrsf12a
Tnfrsf1b
Nfkbia
Nfkbib
Nfkbie
Nfkbil1
Nfkbiz

Figure S7

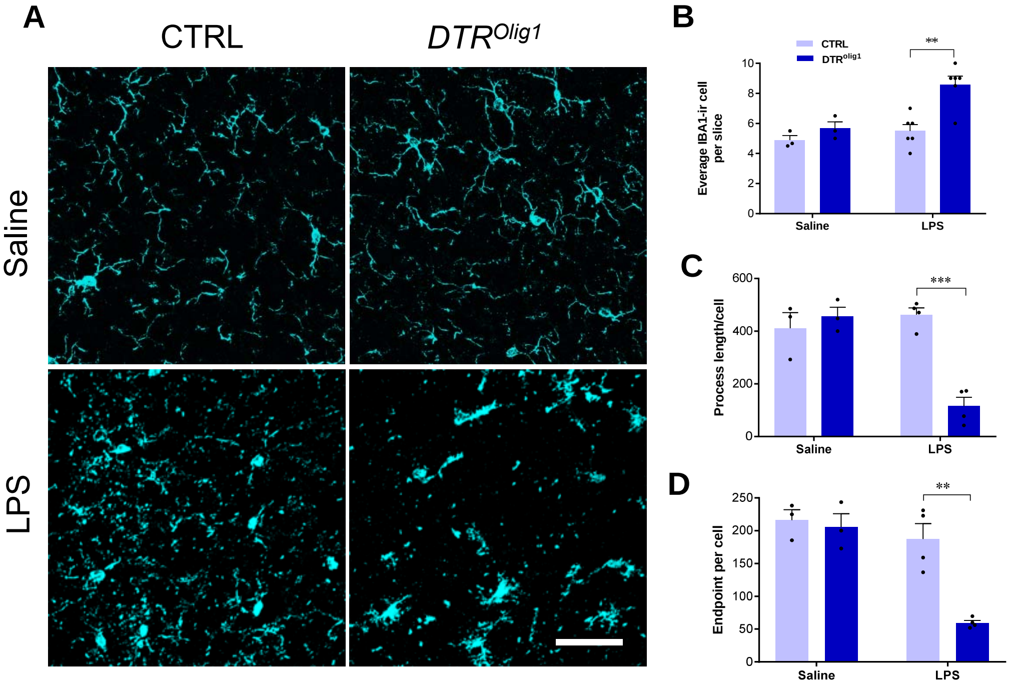
A
CTRL
DTR^Olig1
Saline
LPS
B
CTRL
DTRolig1
**
Everage IBA1-ir cell per slice
10
8
6
4
2
0
Saline
LPS
C
***
Process length/cell
600
400
200
0
Saline
LPS
D
**
Endpoint per cell
250
200
150
100
50
0
Saline
LPS

Figure S8

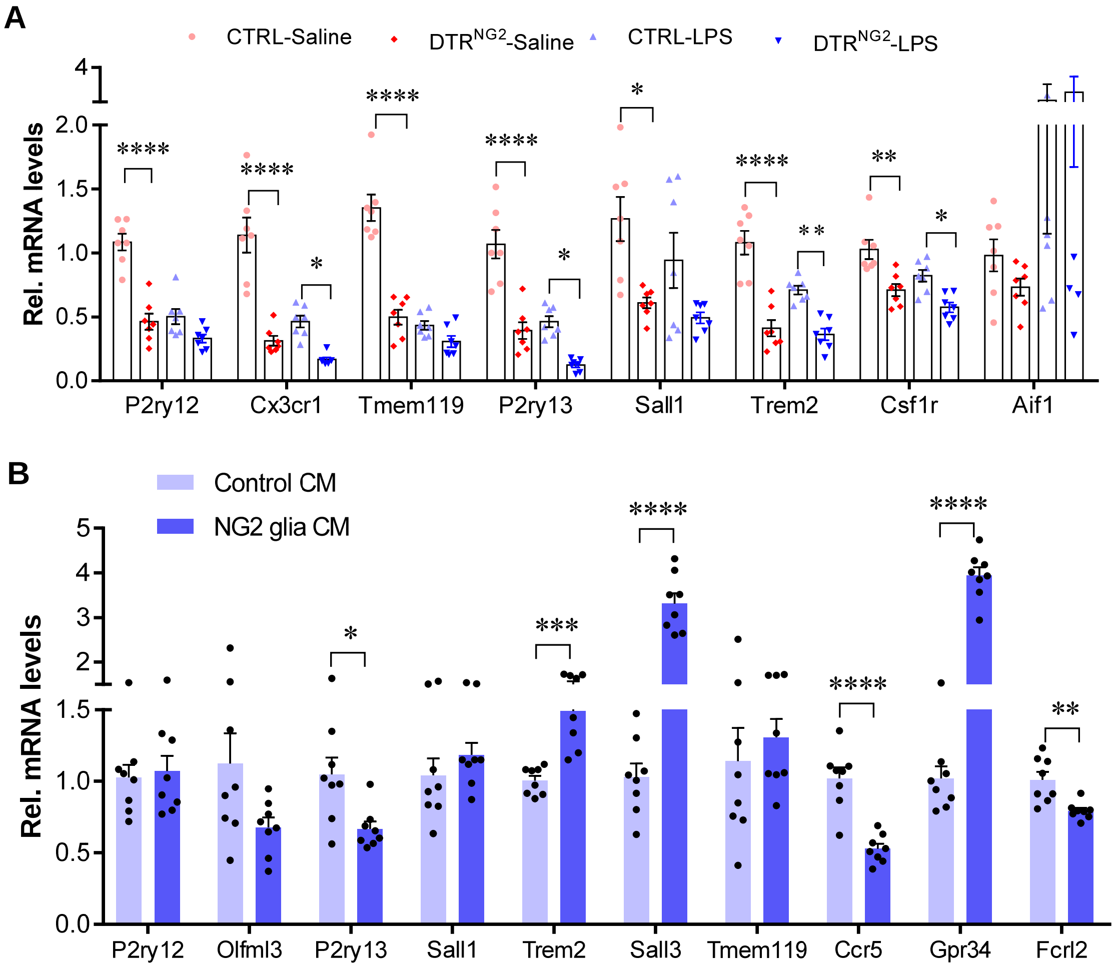
A
CTRL-Saline
DTRNG2-Saline
CTRL-LPS
DTRNG2-LPS
Rel. mRNA levels
4
2.0
1.5
1.0
0.5
0.0
****
****
*
****
****
*
*
****
**
**
*
P2ry12
Cx3cr1
Tmem119
P2ry13
Sall1
Trem2
Csf1r
Aif1
B
Control CM
NG2 glia CM
Rel. mRNA levels
5
4
3
2
1.5
1.0
0.5
0.0
*
***
****
****
****
**
P2ry12
Olfml3
P2ry13
Sall1
Trem2
Sall3
Tmem119
Ccr5
Gpr34
Fcrl2

Figure S9

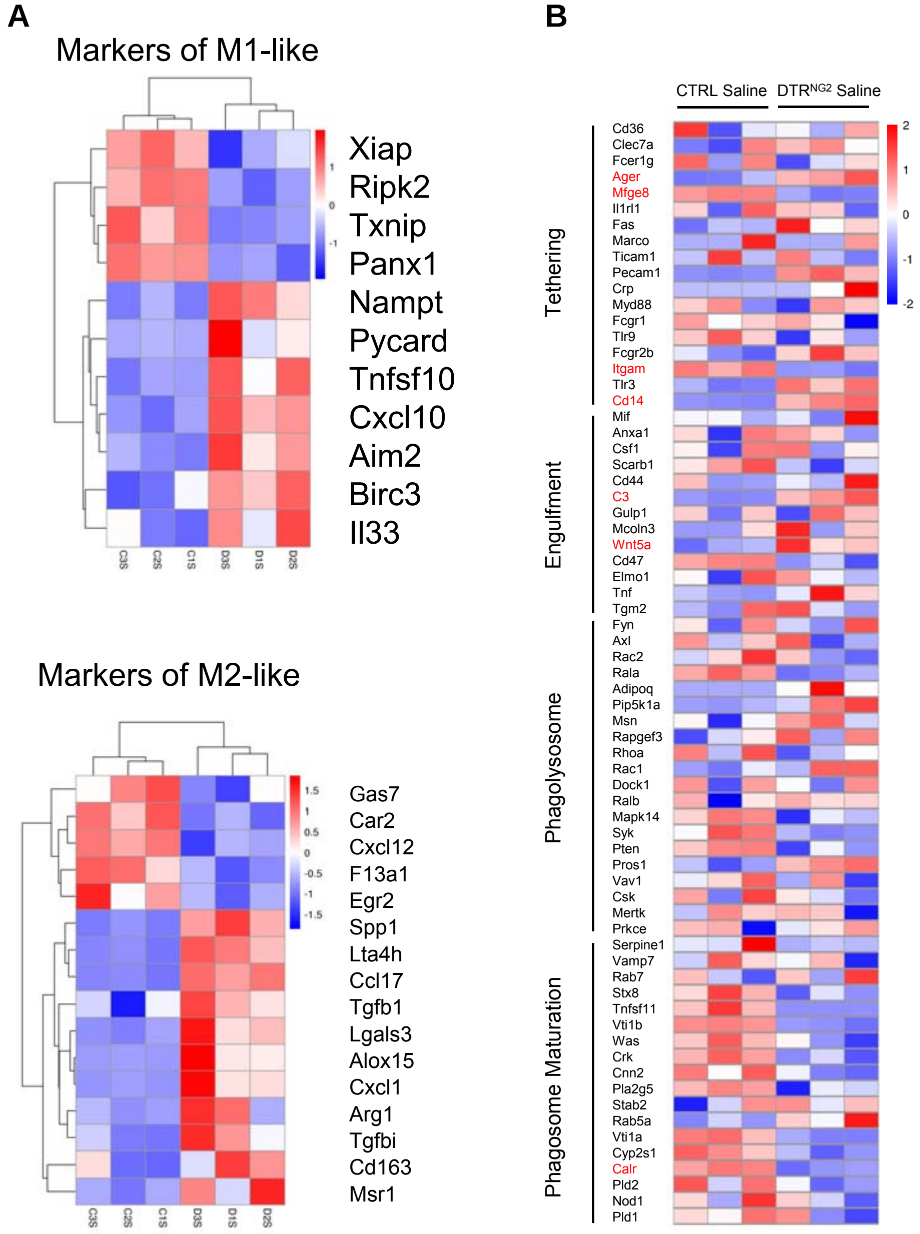
A
Markers of M1-like
Xiap
Ripk2
Txnip
Panx1
Nampt
Pycard
Tnfsf10
Cxcl10
Aim2
Birc3
Il33
C3S
C2S
C1S
D3S
D1S
D2S
Markers of M2-like
Gas7
Car2
Cxcl12
F13a1
Egr2
Spp1
Lta4h
Ccl17
Tgfb1
Lgals3
Alox15
Cxcl1
Arg1
Tgfbi
Cd163
Msr1
B
CTRL Saline
DTRNG2 Saline
Tethering
Engulfment
Phagolysosome
Phagosome Maturation
Cd36
Clec7a
Fcer1g
Ager
Mfge8
Il1rl1
Fas
Marco
Ticam1
Pecam1
Crp
Myd88
Fcgr1
Tlr9
Fcgr2b
Itgam
Tlr3
Cd14
Mif
Anxa1
Csf1
Scarb1
Cd44
C3
Gulp1
Mcoln3
Wnt5a
Cd47
Elmo1
Tnf
Tgm2
Fyn
Axl
Rac2
Rala
Adipoq
Pip5k1a
Msn
Rapgef3
Rhoa
Rac1
Dock1
Ralb
Mapk14
Syk
Pten
Pros1
Vav1
Csk
Mertk
Prkce
Serpine1
Vamp7
Rab7
Stx8
Tnfsf11
Vti1b
Was
Crk
Cnn2
Pla2g5
Stab2
Rab5a
Vti1a
Cyp2s1
Calr
Pld2
Nod1
Pld1

Figure S10

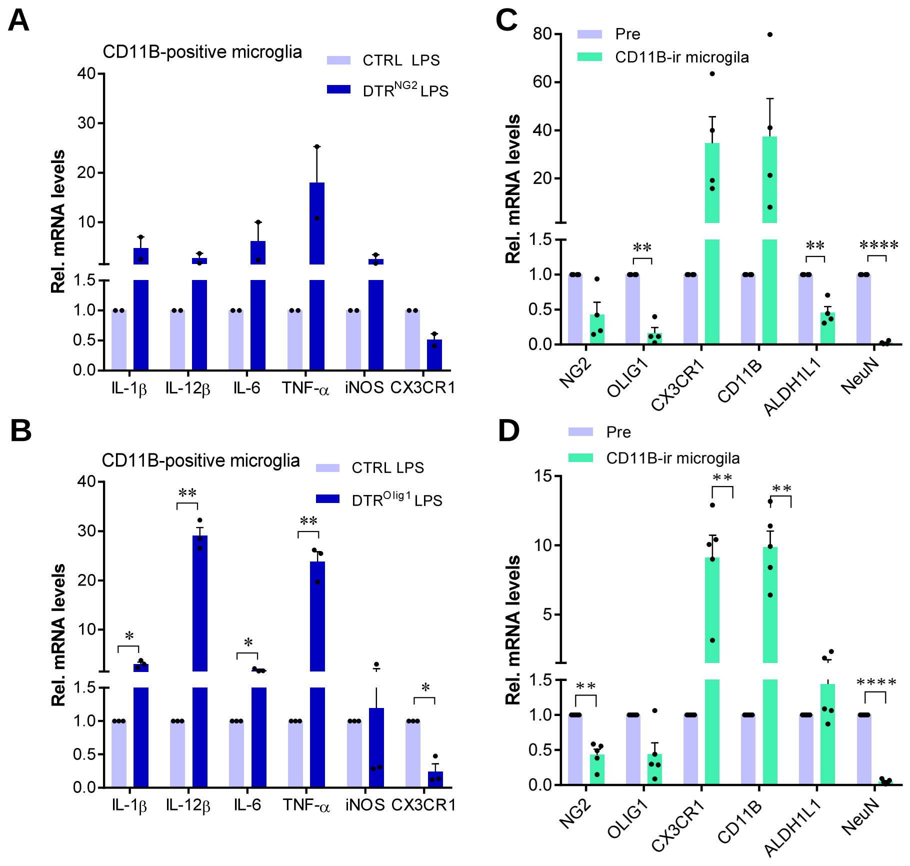

A
CD11B-positive microglia
CTRL LPS
DTR^NG2 LPS
Rel. mRNA levels
IL-1β
IL-12β
IL-6
TNF-α
iNOS
CX3CR1
B
CD11B-positive microglia
CTRL LPS
DTR^Olig1 LPS
Rel. mRNA levels
C
Pre
CD11B-ir microgila
Rel. mRNA levels
NG2
OLIG1
CX3CR1
CD11B
ALDH1L1
NeuN
D
Pre
CD11B-ir microgila
Rel. mRNA levels

Figure S11

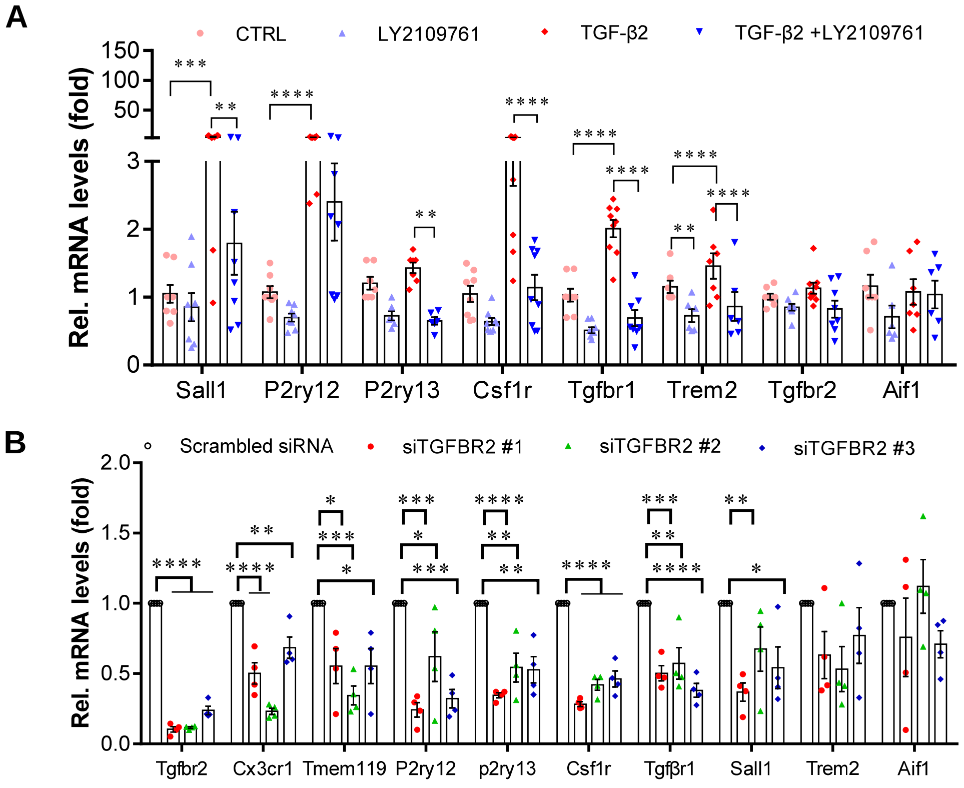
A
CTRL
LY2109761
TGF-β2
TGF-β2 +LY2109761
Rel. mRNA levels (fold)
Sall1
P2ry12
P2ry13
Csf1r
Tgfbr1
Trem2
Tgfbr2
Aif1
B
Scrambled siRNA
siTGFBR2 #1
siTGFBR2 #2
siTGFBR2 #3
Rel. mRNA levels (fold)
Tgfbr2
Cx3cr1
Tmem119
P2ry12
p2ry13
Csf1r
Tgfβr1
Sall1
Trem2
Aif1

Figure S12

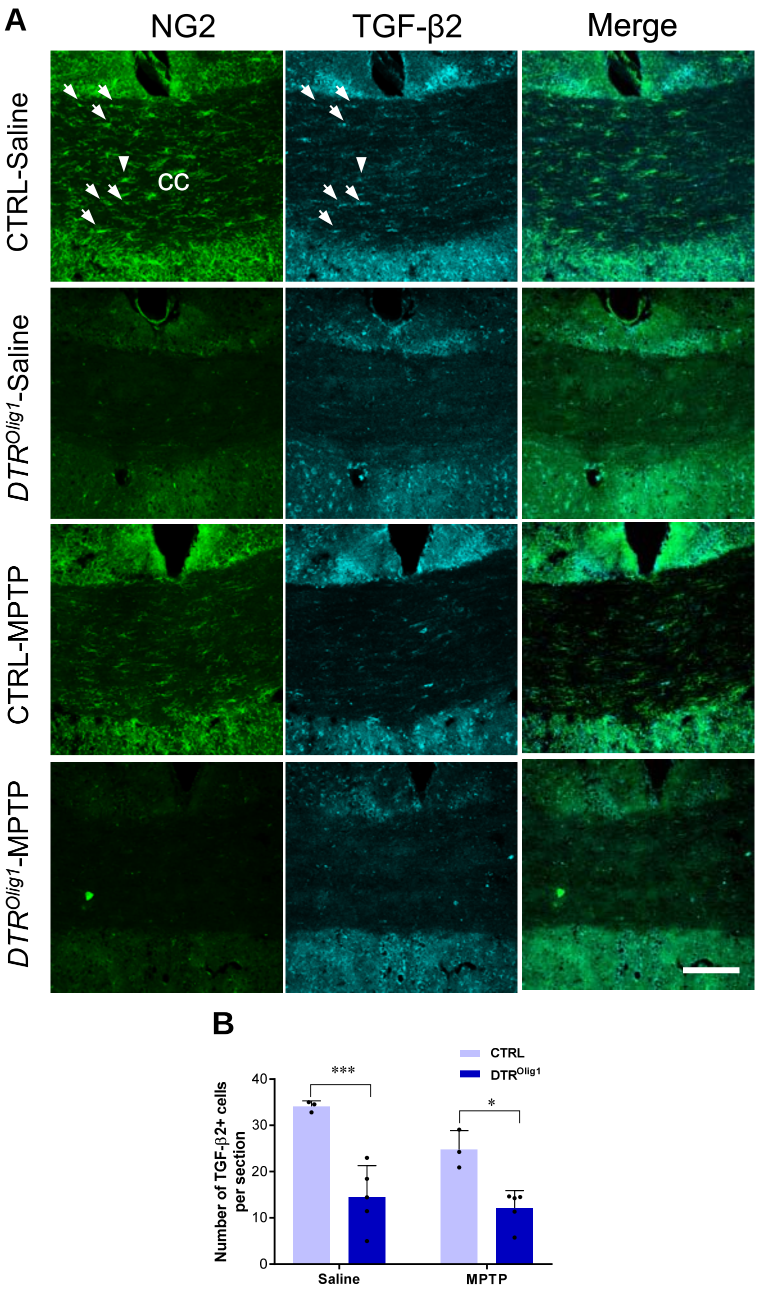

A
NG2
TGF-β2
Merge
CTRL-Saline
DTROlig1-Saline
CTRL-MPTP
DTROlig1-MPTP
CC
B
CTRL
DTROlig1
Number of TGF-β2+ cells per section
40
30
20
10
0
***
*
Saline
MPTP

Figure S13

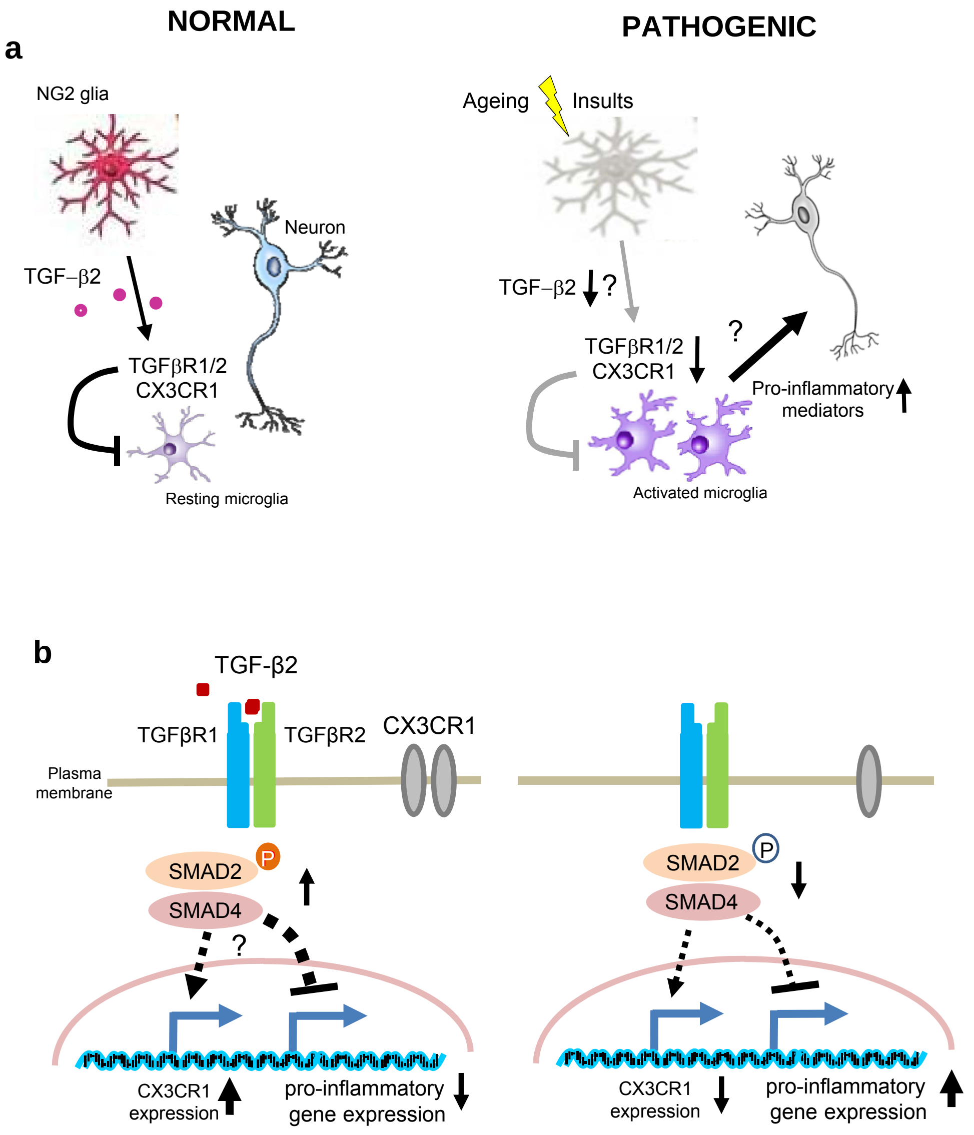
a
NORMAL
PATHOGENIC
NG2 glia
Neuron
TGF–β2
TGFβR1/2
CX3CR1
Resting microglia
Ageing
Insults
TGF–β2 ?
?
Pro-inflammatory mediators
Activated microglia
b
TGF-β2
TGFβR1
TGFβR2
CX3CR1
Plasma membrane
SMAD2
P
SMAD4
?
CX3CR1 expression
pro-inflammatory gene expression
