# Supplementary material for: NG2 glia regulate brain innate immunity via TGF-β2/TGFBR2 axis
Source: BMC Med. 2019 Nov 15;17:204. doi: 10.1186/s12916-019-1439-x (PMC6857135; doi:10.1186/s12916-019-1439-x)
Supplement: Supplementary file 1 — Additional file 1: Supplementary figures. [file 12916_2019_1439_MOESM1_ESM.zip › Legends for supplementary figures_shortened.docx]

**Supplementary Figures**

Figure S1. Efficient ablation of NG2 glia in *DTR^NG2^* mice. **a** Representative micrographs showing the distribution of NG2-positive cells in the indicated brain regions 3.5 days following DT administration. Scale bar, 50 μm. **b** Quantification of NG2 glia after DT injection. Number of NG2-positive cells was counted 3.5 days after DT injection (i.p.). Unpaired two-tailed *t*-test, (n = 4 per group). Unpaired two-tailed t-test, **** *P* < 0.0001.

Figure S2. Acute NG2 glial cell ablation does not impair microvascular integrity in the brain. **a** Photographs showing the brain of *DTR^NG2^* Tg mice and their counterparts following Evens blue dye injection. Lower panel, the brain with ischemia serves as a positive control, whereas intact brain serves as a negative control. **b** The amount of the dye was measured in the brain tissue (**a**). n = 4 per group. Unpaired *t*-test. *P* =0.7532. **c** Immunofluorescent staining of representative sections showing Iectin and IgG immunoreactivities in the cerebrocortex of *DTR^NG2^* Tg mice and littermate controls. No profound IgG immunoreactivity is seen in the brain parenchyma in either *DTR^NG2^* Tg mice and littermate controls. Scale bar, 50 μm. **d** Quantitative data of lectin immunoreactivity shown in (**c**). n = 3 per group. Unpaired *t*-test. *P* > 0.05. **e** qPCR analysis of the striatal tissue of mice shows that there is no significant reduction of PDGFRb mRNA in *DTR^NG2^* Tg mice compared to control. n = 4 per group. Student *t*-test. *P* > 0.05. **f** qPCR analysis shows that there is no significant difference in the levels of Rag1, Lck, IFN-g and CSF mRNA in the cerebrocortical tissue between *DTR^NG2^* mice and littermate controls. All animals received DT injections. Two-way ANOVA with Bonferroni’s post test. (n = 7 - 8 per group: n = 8 for NG2-Cre/saline, *DTR^NG2^* /saline or *DTR^NG2^* /LPS, n = 7 for NG2-Cre /LPS). *P* > 0.05. NS, not significant.

Figure S3**.** Cell-type specificity of NG2 glial cell ablation in *DTR^NG2^* Tg mice. **a** Immunostaining reveals that the distribution pattern of NeuN+ and GFAP+ cells in the cerebrocortex of *DTR^NG2^* Tg mice are not significantly reduced compared to control. Scale bar, 50 μm. **b, c** qPCR analysis of the cerebrocortex of mice shows that there is no significant reduction in mRNA expression levels of NeuN and GFAP in *DTR^NG2^* Tg mice compared to control. (n = 3 -5 per group). Two-way ANOVA. *P* > 0.05. NS, not significant. Scale bars, 50 μm.

Figure S4. Efficacy of Olig1+ cell ablation in the indicated brain regions of *DTR^Olig1^* Tg mice*.* **a-d** Quantification of efficacy of NG2 glia ablation after DT injection. **a** qPCR analysis of *DTR^Olig1^* Tg mice show that there is significant reductions of Olig1 and NG2 mRNA in *DTR^Olig1^* Tg mice following DT administration in both LPS challenged (i.p.) and saline treated groups. Two-way ANOVA with Bonferroni’s post test. (n = 7 - 11). * *P* < 0.05, ** *P* < 0.01, *** *P* < 0.001. **b** Immunofluorescent staining of representative sections showing NG2 immunoreactivities in the indicated brain regions of *DTR^Olig1^* Tg mice and littermate controls. Scale bar, 50 μm. **c** Quantification of NG2+ cells shown in **b**. Unpaired two-tailed *t*-test. (n = 4). **d** Representative immunoblots showing the decreases in the levels of Olig1 protein in *the DTR^Olig1^* Tg mice compared to their counterparts. Unpaired two-tailed *t*-test, (n = 11 - 13) *****P* < 0.0001. **e** Immunofluorescent staining of representative sections showing IgG and lectin immunoreactivities in the cerebrocortex of *DTR^Olig1^* Tg mice and littermate controls. A brain with ischemia serves as positive control. Scale bar, 50 μm. **f** Quantification of data shown in **e**. Data are expressed as mean ± SEM. (n = 4 - 6 per group). Two-way ANOVA with Tukey's multiple comparisons test. *P* > 0.05. **g** Immunostaining analysis of the immunosignal intensities of GFAP (n = 3 per group). *P* > 0.05. Scale bar, 50 μm. **h** Immunofluorescent staining of representative sections showing the immunosignal intensities of NeuN and MBP in Olig1+ cell-ablated mice challenged with vehicle and LPS. **i**, **j** Quantification of data shown in **h**. (n = 4-7). *P* > 0.05. Scale bar, 100 μm.

Figure S5. Efficacy and cell-type specificity of mature oligodendrocyte ablation. **a** Immunofluorescent histochemical staining for CC1 in the indicated brain regions of adult *DTR^Plp1-CreER^* Tg mice and control are shown. Scale bars, 50 mm. **b** Quantitative data shown in **a**. Unpaired two tailed *t* test. (n = 3-6) **P *=* 0.0035 for cerebrocortex, ****P* = 0.0005 for striatum; ***P* = 0.0027 for striatum. **c** qPCR analysis of the hippocampus 1 days after DT administration for the cell specific markers. The levels of NG2, Olig1, MBP, ALDH1L1, CX3CR1 and NeuN were not significantly altered between genotypes. Two-way ANOVA. (n = 3 - 5 per group). *P* > 0.05. **d** Immunofluorescent histochemical staining for NG2 in the indicated brain regions of adult *DTR^Plp1^* Tg mice and control are shown. Scale bars, 200 μm. **e** Quantitative data shown in **d**. (n = 3-4 per group). *P* >0.05.

Figure S6. Gene expression profiles of the brain with ablated NG2 glia alone or combined with LPS challenge using RNA-seq analysis. **a** A graph showing the upregulated expression of inflammation-associated genes belonging to cytokines, chemoattractants, TNF and its receptors and NFκB-associated molecules in *DTR^NG2^* Saline compared to control (*NG2-Cre* SAL). Student *t*-test. (n = 3). **P* < 0.05. **b** Graphs showing relative gene expression levels of cytokines, chemoattractants, TNF and its receptors, NFkB-associated molecules between *DTR^NG2^* LPS and control (NG2-Cre LPS). Student *t*-test, (n = 3). **P* < 0.05, ** *P* < 0.01, *** *P* < 0.001, **** *P* < 0.0001.

Figure S7. Ablation of NG2 glia results in hyper-responsiveness of microglial cells to LPS stimulation. **a** Immunofluorescent histochemical staining for IBA1 in the cerebrocortex of LPS- or saline-treated *DTR^Olig1^* Tg and control mice. Scale bars, 50 μm. **b-d** Quantification of IBA1-positive microglia number (**b**), microglia endpoints/cell (**c**) and process length/cell (**d**). Two-way ANOVA with Tukey's multiple comparisons test, ***P* < 0.01, ****P* < 0.001. n = 3 - 4.

Figure S8. NG2 glia regulates expression levels of microglia-specific gene. **a** NG2 cell ablation results in a significant downregulation of microglia-enriched genes. qPCR analysis of the hippocampus of the *DTR^NG2^* Tg mice and littermate control 4 h after systemic LPS (2.5 mg kg^-1^, i.p.) administration for the cell microglia-enriched genes. These genes including CX3CR1 and P2RY12 displayed a significantly downregulation in the *DTR^NG2^* Tg mice compared with control. **b** Incubation of microglia with conditioned medium derived from NG2 glia elevates the expression levels of microglia-specific genes. Data are expressed as mean ± SEM. Two-way ANOVA with Turkey's post test. (n = 7 mice per group in **a**; n = 7 - 8 in **b**). ** *P* < 0.01, *** *P* < 0.001, **** *P* < 0.0001.

Figure S9. **a** Expression of genes associated with M1/M2 polarization of microglia is perturbed following NG2 cell ablation, as revealed by RNA-seq analysis. Heatmaps showing the gene clusters of M1-like and M2-like markers in the brain of *DTR^NG2^* saline that have 2-fold changes compared to littermate control. **b** A heatmap showing expression of genes involved in the tethering, engulfment, phagolysosome and phagosome maturation of microglia phagocytosis. Genes shown in red are those exhibited 2-fold changes in the brain of *DTR^NG2^* saline compared to control.

Figure S10. Ablation of NG2 glia exacerbates microglia activation in response to LPS challenge. CD11B-positive microglia were isolated from the cerebrocortex of either *DTR^NG2^* or*DTR^Olig1^* Tg mice after the treatment of DT and LPS using MACS. **a, b** Representative graph showing relative mRNA levels of inflammatory mediators derived from microglia isolated from LPS- treated either *DTR^NG2^* (**a**) or *DTR^OLIG1^* (**b**) and their control animals. **c, d** Verification of the identity of isolated microglia from *NG2-Cre* (**c**) or *Olig1-Cre* (**d**) mice. Error bars represent mean ± SEM. (n = 2 for *DTR^NG2^* mice, n = 3 for *DTR^OLIG1^* mice). One-way ANOVA with paired t test. * *P* < 0.05, ** *P* < 0.01, **** *P* < 0.0001.

Figure S11. TGF-β2/TGFBR2 signaling pathway regulates expression of microglia-enriched genes *in vitro.* **a** Representative graph showing the alterations in mRNA expression levels of microglia-enriched genes (Sall1, P2ry12, P2ry13, Csf1r, Tgfbr1, Trem2, TGFBR2) in microglial BV2 cells exposed to either TGF-b2 or TGFBR1/2 inhibitor LY2109761. Two-way ANOVA with Turkey's post test. (n = 6~9 independent experiments). ***P* < 0.01, *****P* < 0.0001. **b** Representative graph showing the alterations in mRNA expression levels of the indicated genes in microglial BV2 cells transfected with three siRNA (siTGFBR2 #1 and siTGFBR2 #2) specific for TGFBR2 for 48 h, separately. One-way ANOVA with Dunnett's post test. (n = 4 independent experiments). **P* < 0.05, ***P* < 0.01, ****P* < 0.001, *****P* < 0.0001.

Figure S12. **a** Immunofluorescent staining of representative sections showing NG2 and TGF-b2 immunoreactivities in the corpus callosum of *DTR^Olig1^* Tg and littermate control 7 days following MPTP exposure. Arrows indicate double-labeled cells. Scale bar, 150 μm. **b** Quantification of NG2-positive cells in the corpus callosum shown in **a**. Two-way ANOVA with Tukey's multiple comparisons test, **P* < 0.05, ****P* < 0.001. n = 3~5.

Figure S13. NG2 glia functions to suppress neuroinflammation by regulation of microglial CX3CR1 expression. **a** A model for the role of NG2 glia in the repression of microglial activation. Under physiological conditions, NG2 glia produces and releases TGF-b2 which is the most abundantly secreted TGF family member among three members examined. TGF-β2 enhances CX3CR1 expression thereby suppressing microglial activation. Under pathological conditions (e.g. brain ageing and neurodegenerative diseases), dysfunctional NG2 glia exhibit a reduction in TGF-β2 levels resulting in a downregulation of CX3CR1 in microglia. This leads to uncontrolled and chronic microglia activation with upregulated pro-inflammatory gene expression. **b** In microglia, TGF-β2 elevates CX3CR1 expression through the classical signaling pathway, and CX3CR1 inhibits LPS-induced neuroinflammation. Under physiological conditions, TGF-β2 may activate microglial TGFBR2 and phosphorylates TGFBR1 which activates SMAD2. The phosphorylated SMAD2 and SMAD4 complex enters into the nucleus and drive CX3CR1 expression. The protein complex also represses the expression of the inflammatory-related genes. Under pathological conditions (e.g. brain ageing and neurodegeneration), dysfunctional NG2 glia exhibit a reduction in TGF-β2 levels resulting in malfunction of SMAD2 and a down-regulation of CX3CR1 in microglia.
